# Structural Insights into SraP-Mediated *Staphylococcus aureus* Adhesion to Host Cells

**DOI:** 10.1371/journal.ppat.1004169

**Published:** 2014-06-05

**Authors:** Yi-Hu Yang, Yong-Liang Jiang, Juan Zhang, Lei Wang, Xiao-Hui Bai, Shi-Jie Zhang, Yan-Min Ren, Na Li, Yong-Hui Zhang, Zhiyong Zhang, Qingguo Gong, Yide Mei, Ting Xue, Jing-Ren Zhang, Yuxing Chen, Cong-Zhao Zhou

**Affiliations:** 1 Hefei National Laboratory for Physical Sciences at the Microscale and School of Life Sciences, University of Science and Technology of China, Hefei Anhui, People's Republic of China; 2 Shanghai Institute of Biochemistry and Cell Biology, Shanghai, China; 3 Center for Infectious Disease Research, School of Medicine, Tsinghua University, Beijing, People's Republic of China; University of California, San Francisco, United States of America

## Abstract

*Staphylococcus aureus*, a Gram-positive bacterium causes a number of devastating human diseases, such as infective endocarditis, osteomyelitis, septic arthritis and sepsis. *S. aureus* SraP, a surface-exposed serine-rich repeat glycoprotein (SRRP), is required for the pathogenesis of human infective endocarditis via its ligand-binding region (BR) adhering to human platelets. It remains unclear how SraP interacts with human host. Here we report the 2.05 Å crystal structure of the BR of SraP, revealing an extended rod-like architecture of four discrete modules. The N-terminal legume lectin-like module specifically binds to N-acetylneuraminic acid. The second module adopts a β-grasp fold similar to Ig-binding proteins, whereas the last two tandem repetitive modules resemble eukaryotic cadherins but differ in calcium coordination pattern. Under the conditions tested, small-angle X-ray scattering and molecular dynamic simulation indicated that the three C-terminal modules function as a relatively rigid stem to extend the N-terminal lectin module outwards. Structure-guided mutagenesis analyses, in addition to a recently identified trisaccharide ligand of SraP, enabled us to elucidate that SraP binding to sialylated receptors promotes *S. aureus* adhesion to and invasion into host epithelial cells. Our findings have thus provided novel structural and functional insights into the SraP-mediated host-pathogen interaction of *S. aureus*.

## Author Summary

*Staphylococcus aureus* is an important pathogen that causes a range of human diseases, such as infective endocarditis, osteomyelitis, septic arthritis and sepsis. The increasing resistance of *S. aureus* to most of the current antibiotics emphasizes the need to develop new approaches to control staphylococcal infections. As a surface-exposed serine-rich repeat glycoprotein (SRRP), *S. aureus* SraP is involved in the pathogenesis of infective endocarditis via its ligand-binding region (BR) adhering to human platelets. However, little is known about how SraP interacts with its host receptor(s). Through structural and functional analyses of the BR domain, we have discovered a specific binding of SraP to N-acetylneuraminic acid (Neu5Ac), in agreement with a recent report of the trisaccharide ligand of SraP. Further mutagenesis analysis showed that SraP binding to Neu5Ac and the trisaccharide promotes *S. aureus* adhesion to and invasion into host epithelial cells. These findings increase our knowledge of surface protein mediated interaction of *S. aureus* with host epithelial cells.

## Introduction

The serine-rich repeat glycoproteins (SRRPs) are a family of adhesins encoded by Gram-positive bacteria that mediate attachment to a variety of host cells or bacteria themselves [1]. SRRPs typically consist of a signal peptide at the N-terminus, a short SRR (SRR1, ∼50–170 residues), a ligand-binding region (BR, ∼250–500 residues) followed by a much longer SRR (SRR2, ∼400–4000 residues), and a C-terminal LPXTG motif anchoring to the cell wall [1]. The BRs of SRRPs from different pathogenic bacteria have varying primary sequences and bind to diverse targets from carbohydrates to proteins [1].

In addition to having highly variable sequences, the BRs from different bacteria are composed of distinct modules. The diversity of BR modules and combinations contributes to the multiple functions of SRRPs. The only four known BR structures to date have identified five distinct modules [2]–[4]. The BR of *Streptococcus parasanguinis* Fap1 contains two modules: an N-terminal helical module and a C-terminal CnaA module [2], whereas *Streptococcus gordonii* GspB has a BR of three modules: CnaA, Siglec and a unique module of unknown function [3]. In addition, the recently reported BR structures of the two SRRP paralogs (Srr1 and Srr2) from *Streptococcus agalactiae* defined two immunoglobulin-fold modules, which specifically bind to the host fibrinogen [4]. However, the module composition and corresponding molecular functions of most BRs remain unknown, which largely impedes the understanding of the pathogenesis mechanism of SRRPs.

*S. aureus* is a human pathogen that causes a wide range of debilitating and life-threatening infections [5]. *S. aureus* encodes a 2,271-residue SRRP termed serine-rich adhesin for binding to platelets (SraP), that is involved in the pathogenesis of infective endocarditis [6]. Moreover, the BR (residues Phe245–Asn751) of SraP, termed SraP_BR_, mediates intraspecies interaction and promotes bacterial aggregation [7]. We determine the 2.05 Å crystal structure of SraP_BR_, revealing a rod-like tandem organization of four discrete modules: a legume lectin-like module, a module with a β-grasp fold, and two tandem cadherin-like modules that create the rigid stem of SraP_BR_. Further structural and biochemical analyses reveal that the legume lectin-like module specifically binds to N-acetylneuraminic acid (Neu5Ac), which may mediate adhesion to host sialylated receptors. These findings increase our knowledge of the diverse BR modules of SRRPs, and provide structural insights into a novel surface protein that mediates interaction of *S. aureus* with host epithelial cells.

## Results

### Overall structure of SraP_BR_

**Figure 1:**
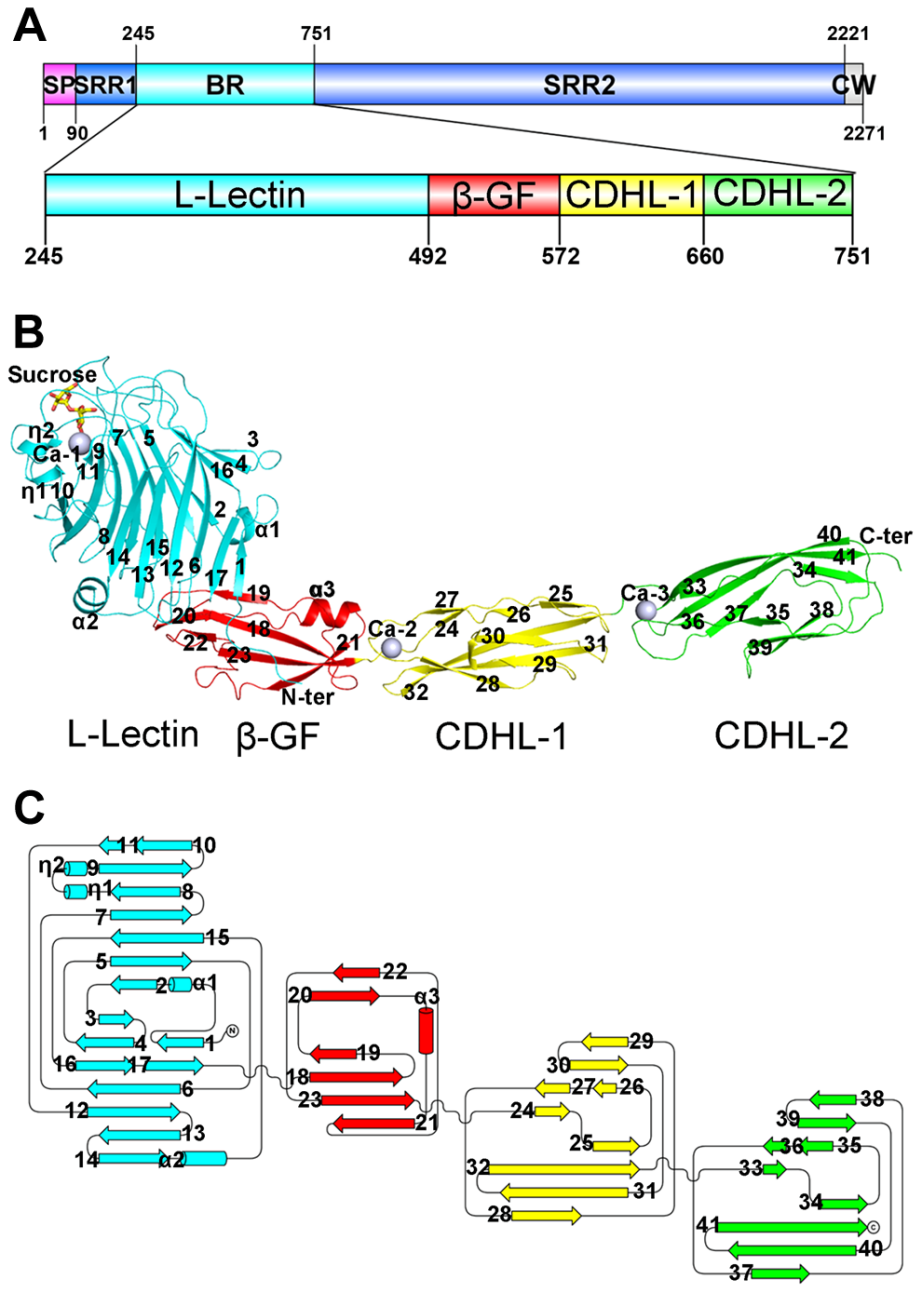
Overall structure of the full-length SraP_BR_. **A**) A diagram to show the organization of SraP and BR. **B**) Cartoon representation of the overall structure of SraP_BR_. **C**) A topology diagram of SraP_BR_. The L-lectin, β-GF, CDHL-1 and CDHL-2 modules are shown in cyan, red, yellow and green, respectively. The sucrose molecule is shown as yellow stick and the bound calcium ions are shown as gray spheres. The secondary structural elements are labeled sequentially.

Each asymmetric unit of the final model at 2.05 Å resolution contains a single SraP_BR_ molecule of residues Thr251–Asn751. The N-terminal residues Phe245–Thr250 are not visible due to their poor electron density. SraP_BR_ folds into a slightly bent, rod-like structure of 160 Å in length that has four discrete modules: a head-like N-terminal module followed a stem of three all-β modules (Fig. 1A). All modules have a dominant β-strand secondary structure. During the model building and refinement process, three peaks of electron density at the 24 σ level were observed in the *|Fo|*−*|Fc|* Fourier difference map, indicating the presence of three metal ions. Atomic absorption spectroscopy assigned these metal ions to Ca^2+^; we thus termed them Ca-1, Ca-2 and Ca-3 accordingly. The structure also contains a sucrose and a 2-(*N*-morpholino)-ethanesulfonic acid (MES) molecule, which were introduced from the cryoprotectant and crystallization buffer, respectively.

The N-terminal module adopts a jelly-roll fold with a β-sandwich (β1–β17) core architecture of two antiparallel β-sheets packed against each other (Fig. 1B). Beyond the core structure, two α-helices (α1 and α2) pack on either side of the lateral of the β-sandwich and partially seal the hydrophobic lateral openings between the two β-sheets. A molecule of sucrose binds to the protruding loops at the distal end of the N-terminal module (Fig. 1B). A structural similarity search using the DALI server [8] revealed that this module is most similar to legume lectins, despite sharing a sequence identity of ≤20%. The top hits include lectins from legume plants such as *Pisum sativum* (PDB 2BQP) [9] and *Robinia pseudoacacia* (PDB: 1FNY) [10], with a Z-score of 22–23 and root mean square deviation (RMSD) of 2.3–2.5 Å over ∼200 Cα atoms. Thus, we termed the N-terminal module L-lectin.

The second module possesses a ubiquitin-like β-grasp fold (β-GF) in the immunoglobulin (Ig)-binding protein superfamily [11]. The module contains a twisted four-stranded mixed β-sheet (β19-β18-β23-β21) packed against a two-stranded antiparallel β-sheet (β20–β22) and an α-helix α3 (Fig. 1B).

The C-terminal tandem repeat of two modules share a sequence identity of 56% and a similar overall structure with an RMSD of 0.81 Å over 81 Cα atoms. The two modules are linked in a head-to-tail fashion, indicating duplication of the coding region during evolution. Each module consists of a β-sandwich of three β-sheets (Fig. 1B & 1C). Structural analysis revealed that the two modules resemble eukaryotic cadherins of known-structure (PDB 3UBF, Z-score 8.6, RMSD 2.3 Å over 85 Cα atoms and PDB 4APX, Z-score 9.5, RMSD 2.3 Å over 87 Cα atoms) [12], [13]. Thus the tandem cadherin-like (CDHL) modules were termed CDHL-1 and CDHL-2, respectively.

### Specific binding of the L-lectin module to N-acetylneuraminic acid

**Figure 2:**
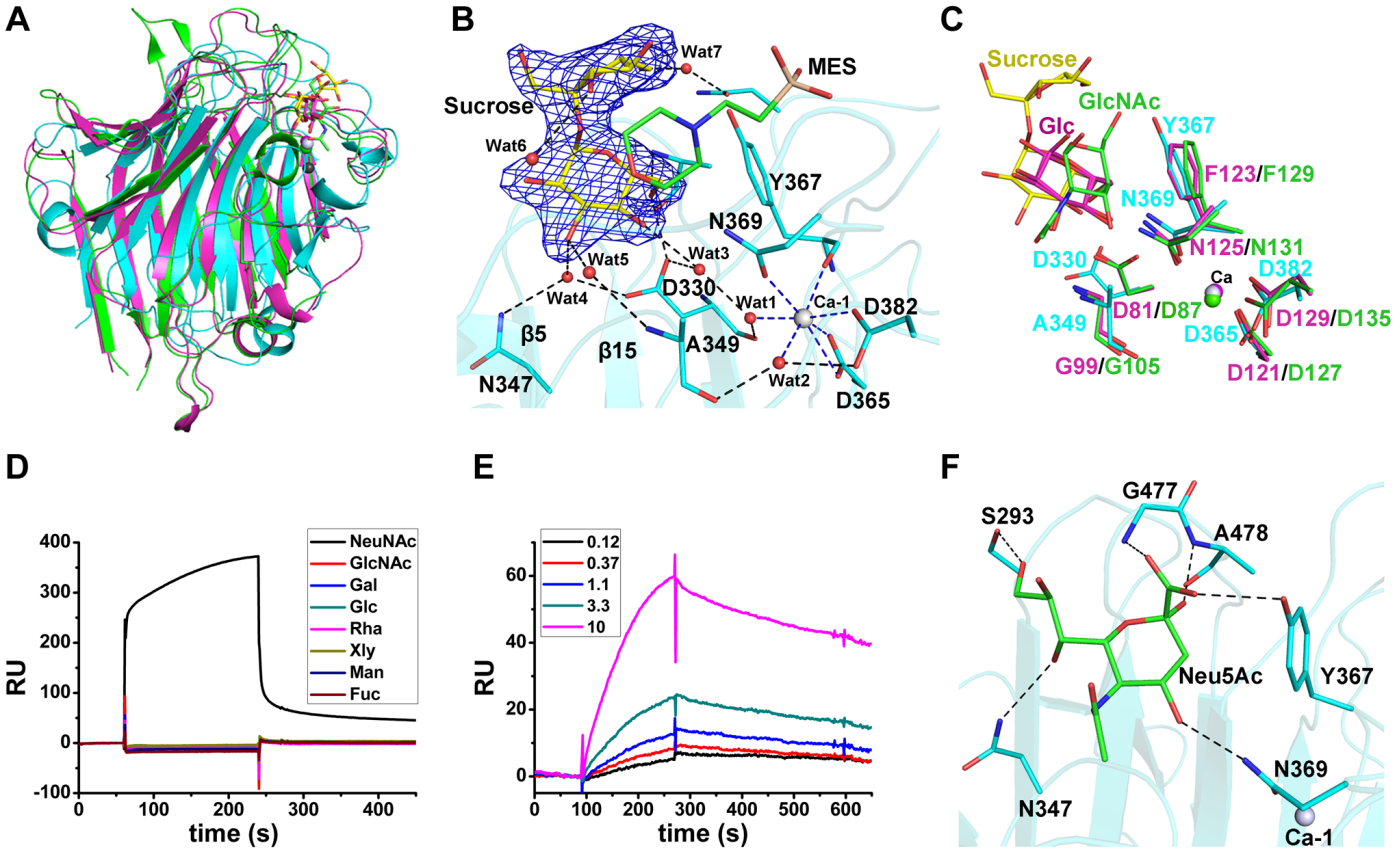
Binding of the L-lectin module to Neu5Ac. **A**) Superposition of the L-lectin module against *P. sativum* pea lectin (PDB 2BQP) and *R. pseudoacacia* bark lectin (PDB 1FNZ). The L-lectin module of SraP_BR_ is colored in cyan, with the carbons of sucrose colored in yellow. The pea and bark lectins are colored in magenta and green, respectively. **B**) The sucrose-binding site and the Ca-1 coordination. The *|Fo|-|Fc|* omit map of sucrose is shown as blue mesh and countered at 3.0 σ. The sucrose and MES molecules and their interacting residues are shown as sticks and colored in yellow, green and cyan, respectively. The water molecules are shown as red spheres and Ca-1 is shown as grey sphere. The polar interactions with sucrose are shown as black dotted lines, whereas the coordinate bonds with Ca-1 are indicated by blue dotted lines. **C**) Comparison of the ligand binding sites of L-lectin module (cyan), pea lectin (magenta) and bark lectin (green). Residues binding to the ligand are shown in sticks. **D**) SPR sensor diagram for the eight tested monosaccharides (D-glucose, D-galactose, D-mannose, L-fucose, N-acetyl-D-glucosamine, N-acetylneuraminic acid, D-xylose, and L-Rhamnose) against the immobilized L-lectin module. **E**) The steady-state equilibrium curve of Neu5Ac at a concentration varying from 0.12 to 10 mM. The binding affinity of the L-lectin module towards Neu5Ac was calculated by using the 1∶1 Langmuir model, the fit of model to the data was determined by an χ^2^ value, and the χ^2^ is 8.98. **F**) A docking model of Neu5Ac (green sticks) binding to the L-lectin module (cyan). Polar interactions are shown in black dashed lines.

The L-lectin module is structurally similar to legume lectins (Fig. 2A), a large family of carbohydrate-binding proteins with diverse activities [14]. Legume lectins commonly coordinate a Ca^2+^ in addition to a transition metal ion, usually Mn^2+^ [15]. In contrast, the L-lectin module has only a Ca^2+^-binding site. At the apex of the L-lectin module, the Ca^2+^ (named Ca-1) was embedded in a 7-coordinate geometry (Fig. 2B). The seven coordinates are from the side-chain oxygen atoms of Asp365 (bidentate coordination from Oδ1 and Oδ2), Asp382 (Oδ2), Asn369 (Oδ1), the main-chain oxygen atom of Tyr367 and two water molecules (Wat1 and Wat2). The two water molecules were further stabilized by the main-chain oxygen atoms of Asp330, Ala349, Oδ1 of Asp382 and another water molecule (Wat3) (Fig. 2B).

A molecule of sucrose is fixed by a cluster of loops protruding from the apex of the L-lectin module. The sucrose molecule has a “bent-back” conformation with the glucose and fructose moieties perpendicular to each other (Fig. 2B). The glucose moiety is inserted in the pocket, and adopts a conformation nearly parallel to the two β-sheet layers. The sugar ring makes hydrophobic interactions with Tyr367 and the morpholine ring of MES, whereas the hydroxyl groups are stabilized by hydrogen bonds with Ala478-Nα, Asp330-Oδ1, and three water molecules (Wat3–5), which are further fixed by residues Asp330, Asn347 and Ala349 (Fig. 2B). In contrast, the solvent-exposed fructose moiety is bent through interactions with MES and two water molecules (Wat6 and Wat7) (Fig. 2B). The sucrose binding residues, especially the stacking residue Tyr367, are structurally conserved in the legume lectins (Fig. 2C).

To identify the favored saccharide of SraP, we first detected the binding affinity of the L-lectin module towards eight common monosaccharides using the surface plasmon resonance assays. Among these monosaccharides, only Neu5Ac bound to the L-lectin module (Fig. 2D). In consequence, we determined that the L-lectin module has an equilibrium dissociation constant (K_d_) of 0.54 mM towards Neu5Ac (Fig. 2E), comparable to previously reported values of legume lectins [16].

Afterwards, we attempted to obtain the Neu5Ac-complexed structure without success. Therefore, we docked Neu5Ac to the structure of the L-lectin module with the sucrose-binding pocket as the search grid. Neu5Ac was docked at a position overlapping the glucose moiety of sucrose, with a shift of ∼1.5 Å towards Ca-1. In the model, Neu5Ac is stacked against Tyr367 via hydrophobic interactions, and makes direct polar interactions with the side chains of Ser293, Asn347, Tyr367 and Asn369 and the Nα atoms of Gly477 and Ala478 (Fig. 2F).

### Structural similarity of the β-GF module to Ig-binding proteins

**Figure 3:**
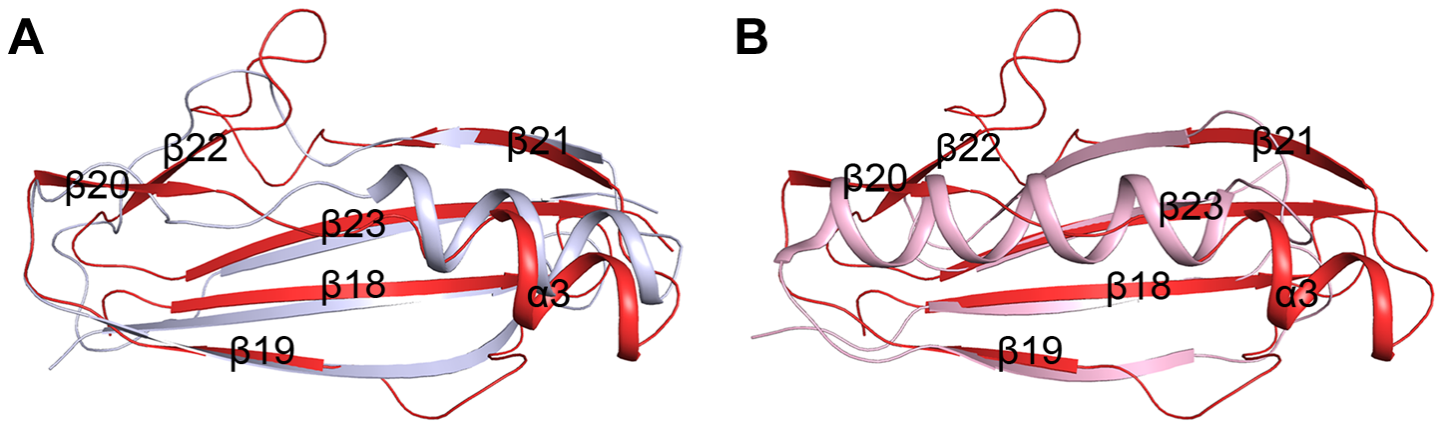
Superpositions of the β-GF module against A) the Ig-binding proteins of B1 domain of mucus-binding protein type 2 repeat Mub-R5 from *L. reuteri* (PDB 3I57), and B) that of Protein L (PpL) from *P. magnus* (PDB 1HEZ). The β-GF module, the B1 domain and PpL are colored in red, grey and light pink, respectively.

The β-GF module adopts a ubiquitin-like β-grasp fold in the Ig-binding superfamily [11]. DALI search [8] suggested the module resembles the B1 domain of mucus-binding protein type 2 repeat Mub-R5 from *Lactobacillus reuteri* [17] (PDB code 3I57, Z-score 9.1, RMSD 1.6 Å, over 68 Cα atoms), and protein L (PpL) from *Peptostreptococcus magnus* [18] (PDB code 1HEZ, Z-score 4.5, RMSD 2.5, over 55 Cα atoms). The B1 domain belongs to a family of Ig-binding proteins [19] that have a core structure of an α-helix packed against a four-stranded β-sheet [17], [20]. The major differences are from the helix α3 and the two lateral β-strands (β19 and β21). In addition, the β-GF module of SraP_BR_ contains two extra β-strands, β20 and β22, which are substituted by loops or α-helix extensions in the B1 domain (Fig. 3). Mub-R5 interacts *in vitro* with a large repertoire of mammalian Ig proteins including secretory IgA, whereas PpL binds to the V_L_ domain of Ig κ chain [19], [21]. Complex structures indicated that formation of a β-zipper is necessary for the binding of PpL to the V_L_ domain of Ig κ chain [18] and IgG or IgM [22]. However, the corresponding β-strands β19 and β21 are much shorter in the β-GF module, which might not be capable of forming a β-zipper.

### The tandem cadherin-like modules create a relatively rigid SraP_BR_ stem

**Figure 4:**
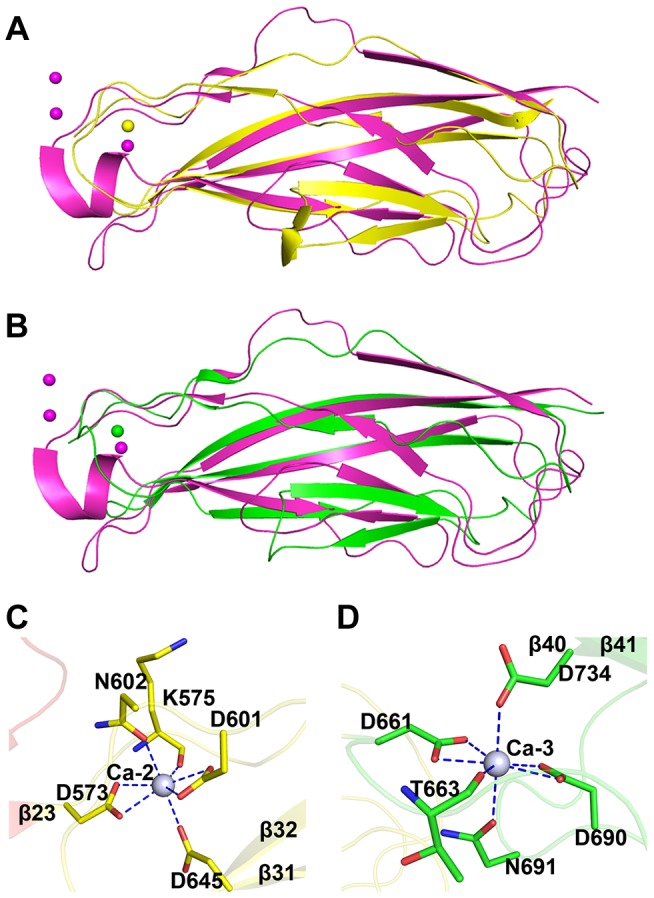
Structural comparison and the calcium coordination patterns of the CDHL modules. Structural comparison of **A**) CDHL-1 and **B**) CDHL-2 to the classic cadherin (PDB 3MVS). The CDHL-1 module, CDHL-2 module and the classic cadherin and corresponding binding calcium ions are shown in yellow, green and red, respectively. The coordination patterns of **C**) Ca-2 and **D**) Ca-3. The coordinate residues are presented in green and yellow sticks, respectively. The coordinate bonds are indicated as blue dotted lines.

The tandem CDHL modules resemble eukaryotic cadherins in a superfamily of calcium-dependent adhesions (Fig. 4A & 4B). Unlike the eukaryotic cadherins, each of which coordinates three Ca^2+^ ions (PDB: 1L3W) [23], CDHL-1 and CDHL-2 binds to Ca-2 and Ca-3 with a 7-coordinate geometry, respectively. Ca-2 is fixed by Asp573 and Asp601 (bidentate coordination from Oδ1 and Oδ2), Asn602 (Oδ1), Asp645 (Oδ2), and the main-chain oxygen atom of Lys575 (Fig. 4C), whereas Ca-3 is coordinated to Asp661 and Asp690 (bidentate coordination from Oδ1 and Oδ2), Asn691 (Oδ1), Asp734 (Oδ2), and the main-chain oxygen atom of Thr663 (Fig. 4D). The coordinate bonds between Ca^2+^ and corresponding residues have a length from 2.2 to 2.6 Å.

**Figure 5:**
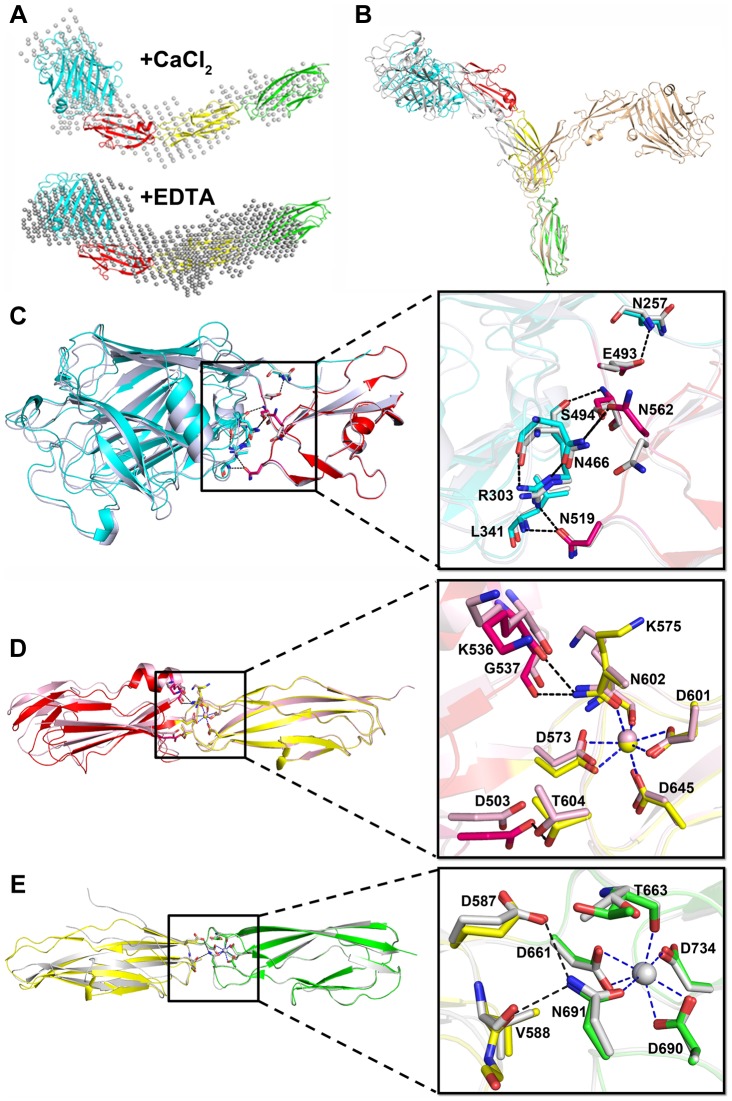
The relative rigid conformation of SraP_BR_ upon binding to calcium. **A**) The SAXS data and envelops for SraP_BR_ in the presence or absence of Ca^2+^. **B**) The final states of the 20-ns molecular dynamics simulation of SraP_BR_ in the presence (gray) or absence (lightpink) of Ca^2+^, compared to the crystal structure of SraP_BR_ with the CDHL-2 modules superimposed. Superposition of **C**) L-lectin&β-GF, **D**) β-GF&CDHL-1 and **E**) CDHL-1&2 to the corresponding modules in the full-length SraP_BR_ structure, respectively. Residues at the interface are shown as sticks, polar interactions are indicated as black dotted lines and the coordinate bonds with Ca-2 and Ca-3 are indicated as blue dotted lines.

The small-angle scattering of X-rays (SAXS) is usually applied to address the flexibility and conformational states of biological macromolecules in solution [24]. To explore the role of Ca^2+^, we used SAXS to compare the overall structure of SraP_BR_ in the presence or absence of Ca^2+^. A difference in wide-angle scattering curves of SAXS indicated that SraP_BR_ adopts different conformations with or without Ca^2+^. Compared to the Ca^2+^-free form, the envelope of Ca^2+^-bound SraP_BR_ correlates much better with the SraP_BR_ crystal structure (Fig. 5A). The larger discrepancies of the Ca^2+^-free SraP_BR_ are mainly resulted from the tandem CDHL modules, indicating that binding of Ca^2+^ makes the tandem CDHL modules more rigid, and thereby facilitates the extended conformation of SraP_BR_ in solution.

We also performed molecular dynamics simulations of SraP_BR_ in the presence or absence of Ca^2+^. Ca^2+^-bound SraP_BR_ remains extended and shows slight conformational changes over the simulation time, whereas removal of Ca^2+^ caused the curling of SraP_BR_ (Fig. 5B). Geometric analysis of the Ca^2+^-coordinating residues revealed larger fluctuations at the two Ca^2+^-binding junctions of Ca^2+^-free SraP_BR_, suggesting the importance of Ca^2+^ for the structural integrity of SraP_BR_. Moreover, the increased RMSD values indicated the two junctions in Ca^2+^-free SraP_BR_ undergo dramatic conformational changes. Given the CDHL-2 modules superimposed, the other three modules adopt a more curved conformation and are projected to the opposite side upon the loss of Ca^2+^ (Fig. 5B).

The rod-like, four-module structure of SraP_BR_ has three junctions with a buried interface of 700, 400 and 220 Å^2^, respectively (Fig. 1B). The interface between L-lectin and β-GF is maintained by several hydrogen bonds including Asn257-Glu493, Arg303-Asn519, Arg303-Ser494, Leu341-Asn519, and Asn466-Asn562 (Fig. 5C). The second interface is formed by residues from β-GF and CDHL-1 via hydrogen bonds of Lys536-Asn602, Gly537-Asn602 and Asp503-Thr604 (Fig. 5D). At the third junction, Glu587 and Val588 of CDHL-1 form two hydrogen bonds with Asn691 of CDHL-2 (Fig. 5E). The second and third interfaces are relatively small; however, they are more stable due to the contribution from coordinate bonds of Ca-2 to Asn602 and Ca-3 to Asn691.

To further investigate the plasticity of these junctions, we determined three crystal structures for each of two consecutive modules: L-lectin&β-GF (Phe245–Lys575), β-GF&CDHL-1 (Ser494–Thr663), and CDHL-1&2 (Ala576–Asn751). We superimposed each of these three structures over the corresponding two modules of the full-length SraP_BR_ structure, always with the C-terminal modules aligned. The results revealed slight twisting and/or translation with intermodule angle changes of 9.4°, 11.7° and 14.6° for the three junctions, respectively (Fig. 5C∼E). In detail, residues Val496-Gln498 in L-lectin&β-GF, Phe571&Thr572 in β-GF&CDHL-1, and Asp661-Thr663 in CDHL-1&2 undergo slight conformational changes. Except for a short disordered segment at the N-terminus of CDHL-1&2 (Fig. 5E) due to the deletion of two Ca-2 coordinate residues Asp573 and Lys575, we did not find secondary-structure change in any module pairs. Moreover, merging these three structures via sequential alignment of the same module resulted in a total twist of 5.4° along the axis of SraP_BR_ structure (Fig. S1), suggesting that the slight interdomain twists of the three junctions are randomly occurred and could be canceled out. Together, the results indicated that in the presence of calcium, SraP_BR_ adopts a relatively rigid rod-like structure under all conditions tested.

### SraP promotes *S. aureus* adhesion and invasion to A549 cells through sialylated receptors

**Figure 6:**
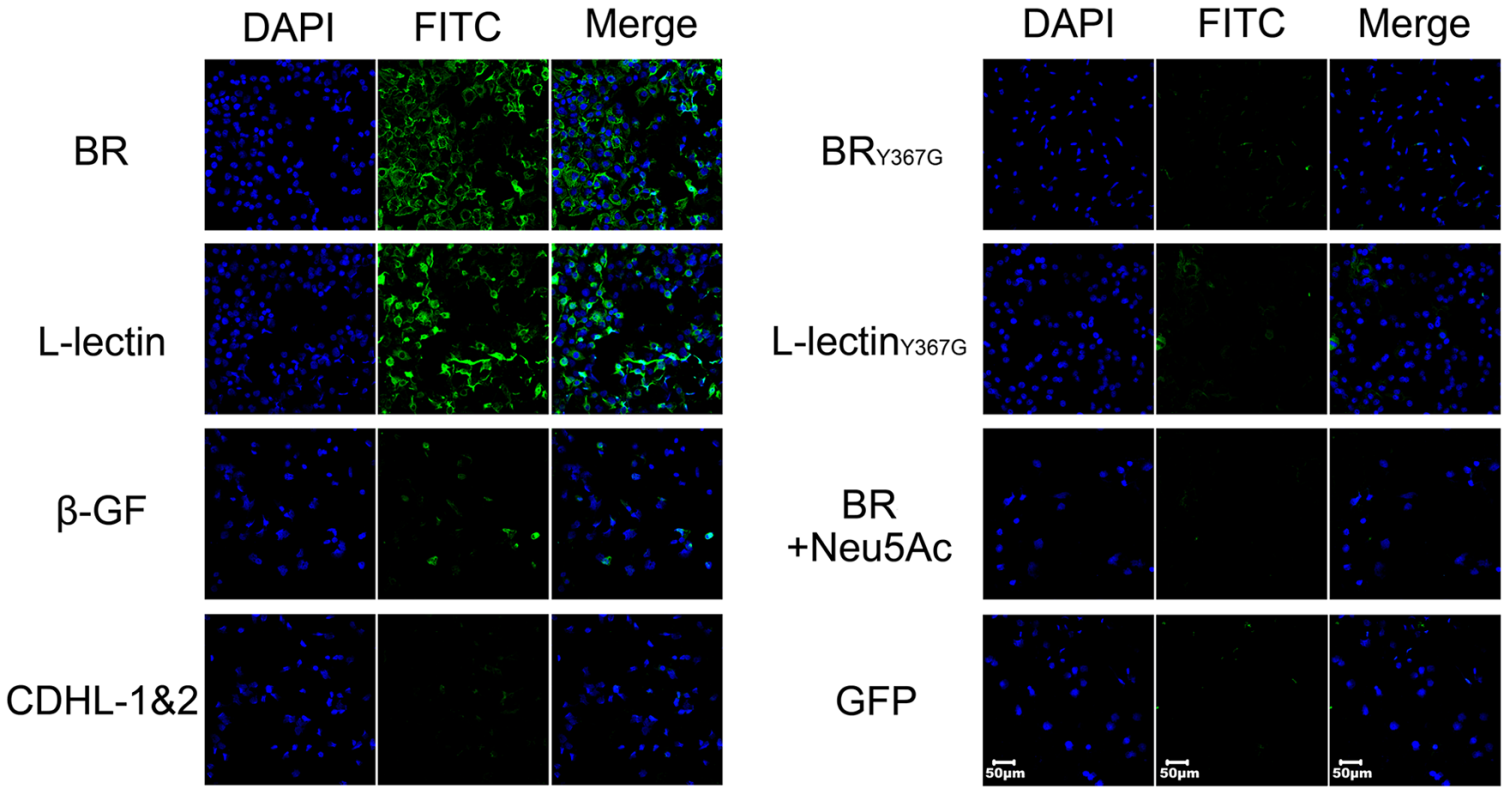
Contribution of the L-lectin module to bacterial adhesion to human lung epithelial cells. A549 cells were incubated with GFP-fused SraP_BR_ (termed BR for short) and truncations (L-lectin, β-GF and CDHL-1&2) or mutants (BR_Y367G_ and L-lectin_Y367G_), respectively. GFP is used as a negative control. The nuclei were stained with DAPI (blue) and the adhered proteins were detected with anti-GFP mouse IgG, followed by FITC conjugated goat anti-mouse IgG (green).

Bacterial attachment and colonization at the surface of host cells have been thought to be mediated by specific binding of BRs to glycoconjugates [1]. It has recently been reported that SraP binds to the salivary agglutinin gp340 via the Neu5Ac moiety of the trisaccharide Neu5Acα(2–3)Galβ(1–4)GlcNAc [25]. In fact, gp340 and homologs are also expressed in lung epithelial cells [26]. To determine if the L-lectin module mediates SraP_BR_ adhesion, we incubated a monolayer of human lung epithelial A549 cells with green fluorescent protein (GFP)-fused SraP_BR_ and individual modules. The results indicated that full-length SraP_BR_ and the L-lectin module, but not the CDHL-1&2 modules or the β-GF module, specifically adhered to the A549 cells (Fig. 6). Tyr367 is a key residue to make hydrophobic interactions with sucrose and the docked Neu5Ac, we thus constructed the Y367G mutant proteins to test the adhesion to A549 cells. As shown in the CD spectra, the mutation of Y367G did not introduce significant structural changes to SraP_BR_ or the L-lectin module (Fig. S2). In contrast, a Y367G mutation in both the full-length SraP_BR_ and the L-lectin module almost completely abolished the adhesion capacity. Moreover, the addition of 5 mM Neu5Ac completely inhibited the adhesion of SraP_BR_ to the A549 cells (Fig. 6). Quantification of the fluorescent signal of three representative frames for each image further confirmed that only the full-length SraP_BR_ and the L-lectin module are capable of specific binding to A549 cells (Fig. S3). These results demonstrate that the adhesion of SraP_BR_ to A549 cells is mediated by the specific recognition of the L-lectin module towards Neu5Ac.

**Figure 7:**
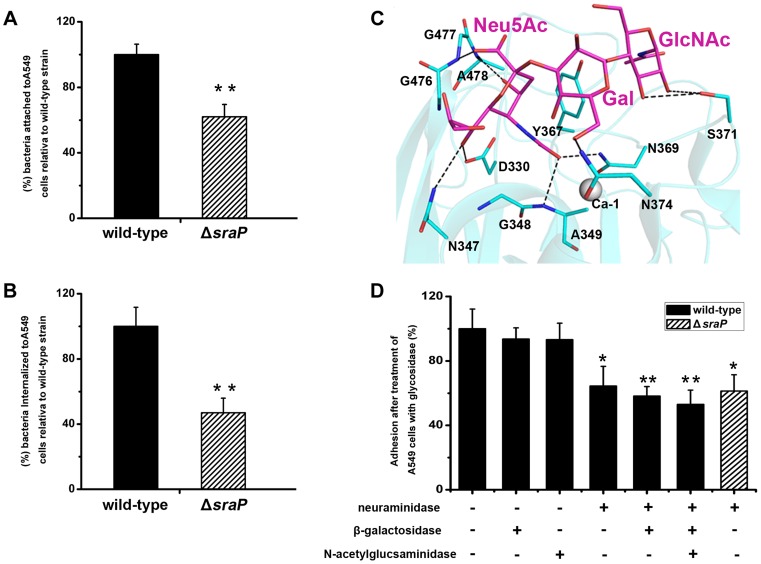
Importance of SraP in bacterial adhesion to and invasion into epithelial cells. Contribution of SraP to the **A**) adhesion and **B**) invasion of *S. aureus* to A549 cells. Experiments were performed in triplicate. Results of representative experiments are presented as mean ± standard deviation. **C**) A docking model of trisaccharide binding to the L-lectin module. The trisaccharide and its interacting residues are shown as sticks and colored in magenta and cyan, respectively. **D**) Adhesion of the wild-type or Δ*sraP* mutant *S. aureus* to A549 cells pre-treated with neuraminidase, β-galactosidase and/or N-acetylglucosaminidase. Experiments were performed in triplicate. The standard error of the mean (SEM) derived from triplicate treatments are indicated as bar graph. All data were normalized against bacterial adhesion to mock-treated cells. Statistical analyses were performed using a one-way ANOVA.

Furthermore we performed comparative assays of bacterial adhesion and invasion to A549 cells using *S*. *aureus* strain NCTC 8325 and an isogenic Δ*sraP* mutant. Deletion of *sraP* resulted in an approximately 40% decrease in adhesion, as compared to the wild-type (Fig. 7A). As a result the level of invasion was decreased by ∼50% (Fig. 7B). These results indicate that SraP contributes to *S. aureus* adhesion to and invasion into host cells.

Together with the recently reported SraP ligand, the trisaccharide Neu5Acα(2–3)Galβ(1–4)GlcNAc [25], our results strongly suggested that the specific binding of SraP_BR_ to the ligand promotes the *S. aureus* adhesion to host cells. We initially tried to determine the complex structure of the trisaccharide with SraP_BR_ or the L-lectin module without success. As an alternative, we docked the trisaccharide to the structure of the L-lectin module (Fig. 7C). In the docking model, the Neu5Ac moiety of the trisaccharide adopts an almost same position to that of Neu5Ac (Fig. 2F). In addition, the moieties of Gal and GlcNAc are stabilized via hydrogen bonds by residues Asn374 and Ser371, respectively (Fig. 7C).

To determine whether the interactions between the L-lectin module and the trisaccharide contribute to the SraP_BR_-mediated adhesion, we used neuraminidase, β-galactosidase or N-acetylglucosaminidase to differentially remove Neu5Ac, Gal, and GlcNAc from the surface of A549 cells (Fig. 7D). Treating A549 cells with neuraminidase alone resulted in an approximately 36% decrease in the adhesion of *S*. *aureus* NCTC 8325. In contrast, the digestion with either β-galactosidase or N-acetylglucosaminidase did not significantly affect the adhesion. Further addition of β-galactosidase and N-acetylglucosaminidase to the neuraminidase-treated A549 cells did not significantly lower the adhesion level (Fig. 7D, the 6^th^ and 7^th^ columns). These results indicated that Neu5Ac is the moiety at the non-reducing end of the trisaccharide, and a major receptor to the SraP-mediated bacterial adhesion of A549 cells. Consistently, the wild-type *S*. *aureus* and Δ*sraP* mutant showed comparable levels of adhesion to the neuraminidase-treated epithelial cells (Fig 7D, the 4^th^ and 8^th^ columns). The adhesion level of the wild-type *S*. *aureus* to the neuraminidase-treated A549 cells (Fig. 7D, the 4^th^ column) is similar to that of the Δ*sraP* mutant to the untreated A549 cells (Fig. 7A). We thus concluded that SraP is the major *S*. *aureus* adhesin that recognizes the sialylated host receptors.

## Discussion

### The specific recognition of the L-lectin module to sialylated receptors may be a universal mechanism for staphylococcal adhesion to host cells

Structural analyses combined with epithelial adhesion experiments demonstrate that SraP plays an important role in mediating bacterial adhesion to host cells by recognizing the L-lectin module. Legume lectins are a large family of proteins primarily found in the seeds of legume plants that have a similar fold but distinct carbohydrate-binding specificities [14]. They typically adopt a quaternary structure of dimers or tetramers that enhances sugar binding specificity or affinity [27]. The architecture of the legume lectin fold has also been found in the animal calcium-dependent lectin ERGIC-53/MR60, a mannose-binding protein involved in the export of soluble glycoproteins from the endoplasmic reticulum [28]. We report here the first legume lectin fold-containing protein in bacteria. This prokaryotic monomeric L-lectin module mediates adhesion to host cells by recognizing Neu5Ac, usually the non-reducing terminal residue of glycoconjugates of extracellular receptors. Bioinformatics analysis suggested that the L-lectin module might also exist in other proteins in *Staphylococci* and *Streptococci* (Fig. S4A). Moreover, the residues involved in Neu5Ac binding are relatively conserved among these putative L-lectins (Fig. S4B). Therefore, we hypothesize that this adhesion mechanism mediated by the L-lectin module operates in other staphylococcal and streptococcal species.

### The L-lectin module is projected outwards mainly by two cadherin-like modules

**Figure 8:**
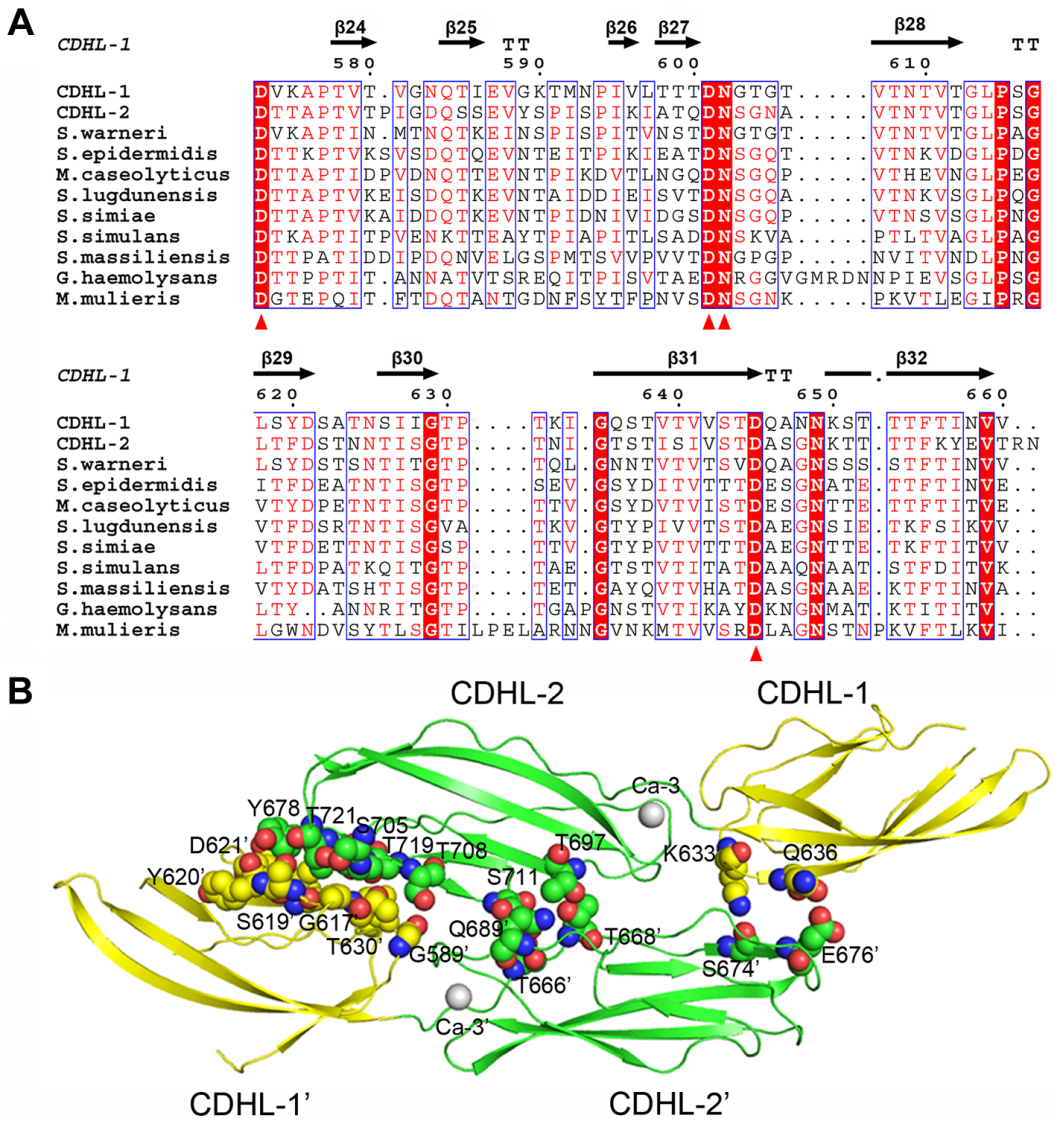
Homodimerization of the tandem CDHL modules. **A**) Multiple-sequence alignment of CDHL modules and their homologs. The Ca^2+^-binding residues are conserved in Gram-positive bacteria. **B**) The dimeric structure of CDHL-1&2. The dimer interface is shown as a CPK model.

To scan the host receptors, the L-lectin module should be projected outwards from the bacterial surface. In addition to a long region of SRR2 that crosses the bacterial cell wall, SraP has a relatively rigid stem of three modules: a β-GF and two CDHL modules. The classic cadherins in vertebrates bridge the intermembrane space between neighboring cells by forming *trans*-adhesive homodimers through membrane-distal extracellular domains [29]. The extracellular domain of most cadherins often contains three conserved Ca^2+^-coordination sites at the interdomain junction [30]. In contrast, each CDHL module in SraP_BR_ binds to only a single Ca^2+^ that does not superimpose on any of the three Ca^2+^ ions in eukaryotic cadherins. Nevertheless, multiple-sequence alignments suggested strong conservation among Gram-positive bacteria of SraP_BR_ Ca^2+^-binding residues (Fig. 8A).

Unlike the monomeric form of SraP_BR_, the recombinant CDHL1&2 exists as a dimer in solution as confirmed by size-exclusion chromatography and chemical cross-linking assays (Fig. S5). The structure revealed that the homodimer of CDHL1&2 buries an interface area of ∼600 Å^2^/per subunit, as calculated by PISA (http://www.ebi.ac.uk/msd-srv/prot_int/cgi-bin/piserver) server [31]. The interface is mainly stabilized by polar interactions, in contrast to the hydrophobic dimeric interfaces of eukaryotic cadherins [29]. CDHL-2 from one subunit packs against the junction between CDHL-1 and CDHL-2 from the symmetric subunit, and vice versa (Fig. 8B). This dimerization pattern may explain the result that SraP mediates intraspecies interaction and promotes aggregation of *S. aureus* ISP479C [7]. However, we did not observe significant decrease of aggregation or biofilm formation upon the deletion of *sraP* in *S. aureus* NCTC 8325. The different results might be due to the variation of *S. aureus* strains. Sequence homology search indicated that some surface proteins in Gram-positive bacteria contain two or more CDHL modules, suggesting that those proteins with multiple CDHL modules have a high potential to mediate intraspecies aggregation.

### SraP_BR_ possesses a unique functional modularization pattern

SRRPs have been identified in a variety of Gram-positive bacteria and function as virulence factors in a wide spectrum of infections [1]. The diversity of these infections (e.g. endocarditis, meningitis and pneumonia) correlates with the variability of BRs. Furthermore, the few BR ligands that have been identified to date range from carbohydrates (such as sialyl-T antigen) [32] to proteins (such as keratins) [33], [34]. In addition to the five distinct modules defined in the four previously reported BR structures [2]–[4], our SraP_BR_ structure identified three types of unique modules. Notably, although the β-GF module resembles the Ig-binding proteins, such as B1 domain of Mub-R5 and PpL [19], [21], the binding of the β-GF module towards human IgG, IgA or IgM could not be detected, in agreement with our structural analysis. We thus propose that the β-GF module, in addition to the two CDHL modules, functions as a relatively rigid stem to project the L-lectin module outwards.

The two structure-known SRRPs, Fap1 and GspB, undergo significant inter-module angle changes, which are regulated by pH and ligand binding, respectively [2], [3]. In contrast, SraP_BR_ appears to adopt a relatively rigid, rod-like conformation when colonizing a host, for the concentration of free Ca^2+^ in the extracellular space or blood is stringently maintained at 1.1–1.3 mM [35]. The rigid architecture of SraP_BR_ enables the globular L-lectin module to extend outwards from the bacterial surface for scanning host receptors. This strategy to expose the functional modules has been observed for other bacterial adhesins such as the fibrillar antigen I/II from *S. mutans* [36], and the rod-like surface protein SasG from *S. aureus* [37].

## Materials and Methods

### Cloning, expression, and purification of SraP_BR_

The genomic DNA from *Staphylococcus aureus* NCTC 8325 was prepared for gene cloning. The DNA sequences (GeneBank, the accession number of YP_501439.1) encoding SraP_BR_ (Phe245–Asn751) and other SraP truncates were cloned into pET28a with an N-terminal His_6_-tag or pET28a with a C-terminal GFP-tag, respectively. The constructs were overexpressed in *E. coli* strain BL21 (Novagen) using LB culture medium (10 g NaCl, 10 g Bacto-Tryptone, and 5 g yeast extract per liter). The cells were grown at 37°C to an OD_600nm_ of 0.6. Expression of the recombinant protein was induced with 0.2 mM isopropyl β-D-1-thiogalactopyranoside (IPTG) at 16°C for another 20 hr before harvesting. Bacteria were collected by centrifugation at 8,000×g for 10 min and resuspended in 30 ml lysis buffer (20 mM Tris-Cl, pH 8.8, 100 mM NaCl). After sonication for 2.5 min followed by centrifugation at 12,000×g for 25 min, the supernatant containing the His-tagged protein was collected and loaded onto a Ni-NTA column (GE Healthcare) equilibrated with the binding buffer (20 mM Tris-Cl, pH 8.0, 100 mM NaCl). The target protein was eluted with 300 mM imidazole, and loaded onto a Superdex 200 column or Superdex 75 column (GE Healthcare; 20 mM Tris-Cl, pH 8.0, 100 mM NaCl). The purity of protein was assessed by electrophoresis and the protein sample was stored at −80°C.

The selenium-Met (SeMet) labeled L-lectin&β-GF protein was expressed in *E. coli* strain B834 (DE3) (Novagen). Transformed cells were grown at 37°C in SeMet medium (M9 medium with 25 µg/ml SeMet and the other essential amino acids at 50 µg/ml) containing 30 µg/ml kanamycin until the OD_600nm_ reached 0.6, and were then induced with 0.2 mM IPTG at 16°C for 20 hr. SeMet substituted protein was purified with the same procedure as the native protein.

The oligomerization state of CDHL-1&2 was analyzed by Superdex 75 column. The protein markers are bovine serum albumin, ovalbumin, chymotrypsinogen A, myoblobulin and ribinuclease A, which have a molecular weight of 67, 44, 25, 17 and 13.7 kDa, respectively (GE Healthcare).

### Crystallization, data collection and processing

All crystals were grown using the hanging drop vapor diffusion method, with a drop of 1 µl protein solution mixed with 1 µl of reservoir solution equilibrated against 500 µl of the reservoir solution. The proteins for crystallization were concentrated by ultrafiltration (Millipore Amicon) to 30, 38, 20 and 20 mg/ml for the full-length SraP_BR_, L-lectin&β-GF, β-GF&CDHL-1 and CDHL-1&2, respectively. The SeMet substituted L-lectin&β-GF protein for crystallization was concentrated to 38 mg/ml. Crystals of SraP_BR_ were grown at 28°C, whereas others were grown at 16°C. Crystals were obtained from 0.8 M (NH_4_)_2_SO_4_ and 0.1 M MES, pH 6.0 for SraP_BR_; 2.5 M sodium formate, 0.1 M sodium acetate pH 4.6 for the native L-lectin&β-GF; 10% PEG 6000, 0.1 M MES, pH 6.0, and 1.0 M lithium chloride for the SeMet substituted L-lectin&β-GF; 2.0 M (NH_4_)_3_PO_4_, 0.1 M Tris-Cl, pH 8.5 for β-GF&CDHL-1 and 1.8 M (NH_4_)_2_SO_4_, 0.1 M HEPES, pH 7.5 for CDHL-1&2. The crystals of SraP_BR_, β-GF&CDHL-1 and CDHL1&2 were transferred to the cryoprotectant with the reservoir solution supplemented with 50% sucrose. The cryoprotectant for the crystals of L-lectin&β-GF consists of the reservoir solution supplemented with 30% glycerol.

All crystals in the cryoprotectant were flash-cooled with liquid nitrogen prior to X-ray diffraction. Data for a single crystal were collected at 100 K in a liquid nitrogen stream using beamline 17U with a Q315r CCD (ADSC, MARresearch, Germany) at the Shanghai Synchrotron Radiation Facility (SSRF). All diffraction data were integrated and scaled with the program HKL2000 [38].

### Structure determination and refinement

The crystal structure of L-lectin&β-GF was determined using single-wavelength anomalous dispersion (SAD) phasing [39] method from a single crystal of SeMet-substituted protein to a maximum resolution of 2.10 Å. The AutoSol program implemented in PHENIX [40] was used to locate the selenium atoms and calculate the phase, which was further improved with the program Buccaneer [41]. Automatic model building was carried out using Autobuild in PHENIX. The initial model was refined in REFMAC5 [42] and Phenix.refine and rebuilt interactively using the program COOT [43]. The model was used as the search model against 2.05 Å SraP_BR_ data by molecular replacement using Molrep program as part of CCP4i [44] program suite. Electron density maps showed clear features of secondary structural elements for automatically building the C-terminal tandem cadherin-like modules using IPCAS [45]. The structures of β-GF&CDHL-1 and CDHL-1&2 were determined by molecular replacement using the corresponding modules in the full-length SraP_BR_ structure as the search model. The initial models were refined by simulated annealing using Phenix.refine to reduce the phase bias. Then the models were refined interactively using COOT and REFMAC5 until the R-factor and R-free values converged. All final models were evaluated with the programs MOLPROBITY [46] and PROCHECK [47]. Crystallographic parameters were listed in Table 1. The *|Fo|-|Fc|* omit map of the sucrose molecule contoured at 3 σ was calculated by FFT implemented in CCP4i. All structure figures were prepared with PyMOL (http://www.pymol.org/).

**Table 1:** Crystal parameters, data collection and structure refinement.

	BR	SeMet- L-lectin&β-GF	β-GF&CDHL-1	CDHL-1&2
**Data collection**				
Space group	*P3_2_21*	*C2*	*P2_1_*	*P3_1_21*
Unit cell				
*a, b, c* (Å)	118.02, 118.02, 136.41;	135.70, 179.64, 71.51;	32.98, 46.46, 47.59;	34.76, 34.76, 237.73;
α, β, γ (°)	90.00, 90.00, 120.00	90.00, 89.77, 90.00	90.00, 102.64, 90.00	90.00, 90.000, 120.00
Resolution range (Å)	50.00–2.05 (2.15–2.05)^a^	50.00–2.10 (2.18–2.10)	50.00–1.60 (1.64–1.60)	50.00–2.24 (2.32–2.24)
Unique reflections	69,226 (6,805)	99,475 (9,904)	18,581 (1,236)	8,890 (866)
Completeness (%)	100 (100)	100 (100)	99.6 (99.1)	99.6 (96.7)
<*I/σ(I)*>	13.8 (5.8)	14.3 (5.6)	10.0 (3.7)	16.7 (8.8)
R_merge_ b (%)	13.7 (38.5)	15.8 (35.7)	10.3 (32.3)	13.1 (38.9)
Average redundancy	6.9 (6.8)	6.9 (6.9)	3.0 (2.6)	8.6 (5.7)
**Structure refinement**				
Resolution range (Å)	50.00–2.05	44.91–2.10	46.44–1.60	50.00–2.24
R-factor^c^/R-free^d^ (%)	18.2/20.5	15.8/19.5	16.4/20.2	27.9/32.6
Number of protein atoms	3,675	9,566	1,251	1,181
Number of water atoms	618	1,448	267	8
RMSD^e^ bond lengths (Å)	0.009	0.011	0.008	0.012
RMSD bond angles (°)	1.285	1.337	1.220	1.569
Mean B factors (Å^2^)	29.5	21.4	21.0	51.9
Ramachandran plot^f^ (residues, %)				
Most favored (%)	98.2	98.8	98.2	97.5
Additional allowed (%)	1.8	1.2	1.8	2.5
Outliers (%)	0	0	0	0
PDB entry	4M00	4M01	4M02	4M03

a The values in parentheses refer to statistics in the highest bin.

b R_merge_ = ∑_hkl_∑_i_|I_i_(hkl)- <I(hkl)>|/∑_hkl_∑_i_I_i_(hkl), where I_i_(hkl) is the intensity of an observation and <I(hkl)> is the mean value for its unique reflection; Summations are over all reflections.

c R-factor  = ∑_h_||Fo(h)|-|Fc(h)||/∑_h_|Fo(h)|, where |Fo| and |Fc| are the observed and calculated structure-factor amplitudes, respectively.

d R-free was calculated with 5% of the data excluded from the refinement.

e Root-mean square-deviation from ideal values.

f Categories were defined by Molprobity.

### Determination of metals binding to the protein

The purified SraP_BR_, CDHL-1&2, BR_Y367G_ and L-lectin_Y367G_ in 20 mM Tris-Cl, pH 8.0, 100 mM NaCl were concentrated to 30, 38, 30 and 21 mg/ml, respectively, and applied to the analyses. Briefly, 500 µl of protein sample was subjected to digestion by the aqueous method using the HNO_3_ and HClO_4_ (4∶1, v/v) method. Afterwards, the digested samples were diluted with deionized water and analyzed by atomic absorption spectroscopy (Atomscan Advantage, Thermo Ash Jarell Corporation, USA).

### Surface plasmon resonance (SPR) assays

The binding affinities of the L-lectin module towards varying monosaccharides were determined by SPR. SPR experiments were performed at 25°C using a Biacore 3000 instrument using HBS (10 mM HEPES, pH 7.5, 150 mM NaCl) containing 0.005% (v/v) Tween 20 and a flow rate of 5 µl/min. The L-lectin module was covalently immobilized on the carboxymethyldextran surface of the CM5 chip. The chip was activated with EDC (N-ethyl-N-[3-dimethylaminopropyl] carbodi-imide)/NHS (N-hydroxysuccinimide) solution, and the L-lectin module in 10 mM acetate buffer (pH 5.5) was injected into the flow channel. At the end, the sensor surface was blocked with 1 M ethanolamine. The blank channel was treated in the same way without protein injected. Each monosaccharide in the running buffer was incubated for 1 min in the flow-cells using the kinject mode. Both injection and dissociation steps last for 5 min. The sensor surface was regenerated with 50 mM NaOH. All analyses were performed with the BIAeval software. The equilibrium responses were plotted versus monosaccharide concentrations and fitted to a 1∶1 Langmuir binding model using the Origin 8.0 software (OriginLab Corp.).

### Computational docking

The docking of Neu5Ac or the trisaccharide Neu5Acα(2–3)Galβ(1–4)GlcNAc to the L-lectin module of SraP_BR_ was performed with AutoDock Vina software (version 1.0) [48], which uses a unique algorithm that implements a machine learning approach to its scoring function. This docking allowed a population of possible conformations and orientations for the ligand at the binding site to be obtained. Using AutoDock Tools (ADT) 1.5.4 [49], polar hydrogen atoms were added to the L-lectin structure, and its non-polar hydrogen atoms were merged. The protein and ligands were converted from a PDB format to a PDBQT format. All single-bonds within Neu5Ac were set to allow rotation. A grid box covering the entire sucrose-binding site was used to place Neu5Ac freely. The results were sorted by binding affinity and visually analyzed using PyMOL.

### Chemical cross-linking

Chemical cross-linking of purified CDHL-1&2 was performed using formaldehyde and bis(sulfosuccinimidyl) suberate (BS^3^), which is a homobifunctional sulfo-N-hydroxysuccinimide ester analog with a spacer arm length of 1.14 nm (Pierce). Briefly, for formaldehyde cross-linking assays of CDHL-1&2, 20 µl of the recombinant protein (2 mg/ml) was mixed with 20 µl PBS containing 2% formaldehyde, and the samples were incubated at 25°C for 30 min and 1 hr, respectively. For BS^3^ cross-linking assay of CDHL-1&2, 100 µl recombinant protein (2 mg/ml) was incubated with 5 mM BS^3^ at 25°C for 30 min and 1 hr, respectively. The reaction was quenched by the addition of 20 mM Tris-HCl, pH 8.0. Then the samples were separated by 10% SDS-PAGE and were stained by Coomassie Brilliant Blue G250. Molecular mass markers for SDS-PAGE were purchased from Thermo Scientific (Wilmington, DE): beta-galactosidase, bovine serum albumin, ovalbumin, lactate dehydrogenase, REase Bsp98I, beta-lactoglobulin, lysozyme, which have a molecular weight of 116, 66.2, 45, 35, 25, 18.4 and 14.4 kDa, respectively.

### Bacterial strains, media and growth conditions

*Staphylococcus aureus* NCTC 8325 and its derivative strains were grown in LB medium, and when necessary, erythomycin (2.5 mg/ml) and chlorampenicol (15 mg/ml) were added. To generate the insertion-deletion mutagenesis, approximately 500 bp of upstream and downstream fragments of the BR region of *sraP* gene was amplified by polymerase chain reaction (PCR), digested with *Pst*I/*Sal*I and *Bam*HI/*Xba*I, respectively, and then ligated to either end of a double-digested (*Bam*HI/*Sal*I) erythromycin-resistance gene (Erm^R^), which was amplified from plasmid pEC1. The three fragments were ligated with the erythromycin-resistance gene in the middle, and then cloned in the temperature-sensitive shuttle vector pBT2. The resulting plasmid was transformed by electroporation into *S. aureus* strain RN4220 for propagation, and then transformed into *S. aureus* NCTC 8325 for allelic exchange. Mutants were screened and further checked by PCR and sequencing.

### Cell adhesion and invasion assays

Adhesion and invasion experiments were performed as described previously [50]. A549 human respiratory epithelial cells were grown in Dulbecco's modified Eagle's medium (DMEM, Gibco) supplemented with 10% fetal bovine serum (FBS), 5 mM glutamine, penicillin (5 µg/ml) and streptomycin (100 µg/ml). Approximately 5×10^5^ cells were seeded into 24-well tissue culture plates, and allowed to grow in 5% CO_2_ at 37°C. Before use, the monolayers were washed twice with PBS. For adhesion assays, *S. aureus* grown to OD_600nm_ of 0.5 in LB medium were resuspended in cell culture medium without serum. Bacteria were diluted to a concentration of 2×10^7^ CFU/ml and were used to infect the confluent cell monolayers at 37°C for 1 hr. After incubation, the infected monolayers were washed five times with PBS to remove non-adhered bacteria, and treated with 200 µl trypsin (2.5 mg/ml) at 37°C for 3 min to release the adhered bacteria. A549 cells were lysed with 0.05% Triton X-100. The number of adhered bacteria was determined by plating serial dilutions of the recovered bacterial suspensions onto LB agar. For the deglycosylation of A549 cells, The cells were incubated with DMEM media containing 0.008 units ml^−1^ purified *Clostridium perfringens* neuraminidase (Sigma), 40 nM purified β-galactosidase (Spr0565) from *S. pneumoniae* or 0.0025 units ml^−1^ of *S. pneumoniae* β-N-acetylglucosaminidase (Sigma) at 37°C for 4 hr in 5% CO_2_ [34].

For invasion assays, the bacteria in each well, after incubated in 0.5 ml DMEM for 1 hr, were incubated in 1 ml fresh DMEM containing 14 µg/ml of gentamicin (Sigma) for another 1 hr. Cell monolayers were washed three times with sterile PBS and lysed with 0.05% Triton X-100. The internalized bacteria were counted by plating serial dilutions of the recovered bacterial suspensions onto LB agar. Experiments were performed in triplicate. Data corresponding to adhesion and invasion were compared using the Mann-Whitney tests. Statistical differences were determined with the t-test.

### Immunofluorescence assays

Immunofluorescence assays were performed as described previously [51]. Briefly, GFP and GFP-fused proteins at 10 µM were suspended in the media in the absence of serum and antibiotics, and incubated on ice for 2 hr with the monolayer of A549 cells grown on the 12-mm glass coverslips. For inhibition assays, Neu5Ac at 5 and 10 mM was pre-incubated with GFP-fused SraP_BR_ protein, respectively. After incubation, cells were sequentially washed three times with cold PBS, fixed with 4% paraformaldehyde for 10 min, permeabilized with 0.1% Triton X-100 for 2 min, and incubated with GFP-tag mouse antibody and a fluorescein isothiocyanate (FITC)-conjugated goat anti-mouse IgG antibody. The nuclei were stained with 4′,6-diamidino-2-phenylindole (DAPI) reagent. Slides were examined with a Zeiss LSM710 confocal scanning fluorescence microscope (Carl Zeiss, Jena, Germany) with a Plan-Apocromat 20×/0.8NA objective. Confocal parameters set for immunofluorescence detection were taken as standard settings. The excitation wavelength is 405 nm and the emission wavelength is 410–492 nm. The confocal images were collected and processed with the software ZEN 2009. Experiments were performed in triplicate, with three or more replicate wells tested for each experimental condition.

### Molecular dynamics simulations

Molecular dynamics simulations were performed for SraP_BR_ in the Ca^2+^-bound and the Ca^2+^-free states, respectively. For each simulation, the system was placed in a TIP3P [52] water box with a distance of at least 12 Å to the box boundaries. Ions were added to neutralize the system and to result in a concentration of 0.15 M NaCl. The solvated protein was subjected to energy minimization employed the steepest descent algorithm and conjugate gradient, respectively. Simulations were performed with a parallel implementation of the GROMACS (version 4.5.5) package [53] using the AMBER03 force field [54]. MD productions were run for 20 ns using a time step of 2 fs and the NPT ensemble [55]. Covalent bonds were constrained using the LINCS algorithm [56], while the cutoff distances for the Coulomb and van der Waals interactions were set to 0.9 and 1.4 nm, respectively. The long-range electrostatic interactions were treated by the PME algorithm [57] with a tolerance of 10^−5^ and an interpolation order of 4. Structure visualization was performed with VMD [58].

### Small-angle X-ray scattering (SAXS) experiments

SAXS was used to investigate the overall conformations of SraP_BR_ in the presence or absence of calcium. The full-length SraP_BR_ at 1.0 and 5.0 mg/ml was analyzed, either in 10 mM calcium chloride or in 50 mM EDTA. SAXS data were collected on the 12ID beamline of Advanced Photon Sources (APS) at the Argonne National Laboratory using the Pilatus 2M detector (DECTRIS, Switzerland). The scattering patterns were measured with a 1–2 second exposure time for each collected frame, and twenty frames were taken for each sample to optimize the signal-to-noise ratio. To reduce the radiation damage, a flow cell made of a cylindrical quartz capillary with a diameter of 1.5 mm and a wall of 10 µm was used during the data collection process. No concentration effect was observed. All SAXS curves were measured at the room temperature over the range of momentum transfer 0.006<s<0.82 Å^−1^ (where s = 4π sin(θ)/λ, 2θ is the scattering angle, and the X-ray wavelength λ is 1.033 Å). The data were processed using the PRIMUS [59] program package and standard procedures. The forward scattering (*I(0)*) and the radius of gyration (*R_g_*) were evaluated using the Guinier approximation assuming that at very small angles (s<1.3/*R_g_*) the intensity is represented as I(s)  =  *I(0)*exp(−(s*R_g_*)^2/3^).

The program GNOM [60] was used to calculate D_max_ and the interatomic distance distribution function *p(r)*. The particle shape of each measured sample was reconstructed *ab initio* using the programs DAMMIN [61] and GASBOR [62]. The scattering patterns of the atomic crystal structures for SraP were calculated using the program CRYSOL. For the *ab initio* analyses and modeling, multiple runs were performed to verify the stability of the solution.

## Supporting Information

Figure S1 The intermodule twist along the axis of SraP_BR_. The merged structure of SraP_BR_ (in orange) was generated by sequentially superimposing the same module against each other from the three structures (L-lectin&β-GF, β-GF&CDHL-1 and CDHL-1&2). The L-lectin, β-GF, CDHL-1 and CDHL-2 modules of SraP_BR_ are shown in cyan, red, yellow and green, respectively. The merged SraP_BR_ was superimposed against the crystal structure of SraP_BR_ with the two CDHL-2 modules aligned.
(TIF)

Figure S2 The CD spectra of **A**) SraP_BR_ and **B**) the L-lectin module. The results demonstrated that mutation of Y367G did not introduce significant changes to the protein structures.
(TIF)

Figure S3 Quantitation of the GFP fluorescent signals of SraP_BR_ and mutants. The fluorescent signals for each protein were quantified by calculating the mean gray values of three representative frames using the ImageJ software (http://imagej.nih.gov/ij/). The average mean gray value and standard error of the mean (SEM) derived from triplicate treatments are indicated as bar graph.
(TIF)

Figure S4 The L-lectin module is conserved in *Staphylococci* and some species of *Streptococci*. **A**) Schematic of *Staphylococcal* and *Streptococcal* proteins containing an L-lectin module. The first line represents the SraP protein from *S. aureus*. Similar modularization is observed in *S. epidermidis* and *S. warneri* (2^nd^ and 3^rd^ line). The 4^th^ line represents uncharacterized protein from *S. salivarius*. The 5^th^ is a serine threonine rich antigen from *S. canis*, and the last is a putative uncharacterized protein from *S. australis*. SP: signal peptide, CWA: cell wall anchor motif, Rib: a repeat motif in Rib protein of group B *Streptococcus*, Ig/albumin-bd: immunoglobulin and albumin-binding domain. **B**) Multiple-sequence alignment of the L-lectin module and homologs. The Neu5Ac binding residues (red triangle) are conserved.
(TIF)

Figure S5 CDHL1&2 exists as a dimer in solution. **A**) The size-exclusion chromatograph profile of CDHL-1&2. The standard curve was inserted as an inlet. Chemical cross-linking of the purified CDHL-1&2 proteins using **B**) formaldehyde (FA) and **C**) bis(sulfosuccinimidyl) suberate (BS^3^). The protein samples were separated by 10% SDS-PAGE. The bands corresponding to the monomer and the dimer of CDHL1&2 are labeled.
(TIF)

## References

[1] Lizcano A, Sanchez CJ, Orihuela CJ (2012) A role for glycosylated serine-rich repeat proteins in Gram-positive bacterial pathogenesis. Mol Oral Microbiol 27: 257–269. 22759311 10.1111/j.2041-1014.2012.00653.x PMC3390760

[2] Ramboarina S, Garnett JA, Zhou M, Li Y, Peng Z, et al (2010) Structural insights into serine-rich fimbriae from Gram-positive bacteria. J Biol. Chem 285: 32446–32457. 10.1074/jbc.M110.128165 PMC2952246 20584910

[3] Pyburn TM, Bensing BA, Xiong YQ, Melancon BJ, Tomasiak TM, et al (2011) A structural model for binding of the serine-rich repeat adhesin GspB to host carbohydrate receptors. PLoS Pathog 7: e1002112. 21765814 10.1371/journal.ppat.1002112 PMC3131266

[4] Seo HS, Misanov G, Seepersaud R, Doran KS, Dubrovska I, et al (2013) Characterization of fibrinogen binding by glycoproteins Srr1 and Srr2 of *Streptococcus agalactiae* . J Biol Chem 288: 35982–96. 24165132 10.1074/jbc.M113.513358 PMC3861647

[5] Lowy FD (1998) Staphylococcus aureus infections. N Engl J Med 339: 520–532. 9709046 10.1056/NEJM199808203390806

[6] Siboo IR, Chambers HF, Sullam PM (2005) Role of SraP, a serine-rich surface protein of *Staphylococcus aureus*, in binding to human platelets. Infect Immun 73: 2273–2280. 15784571 10.1128/IAI.73.4.2273-2280.2005 PMC1087419

[7] Sanchez CJ, Shivshankar P, Stol K, Trakhtenbroit S, Sullam PM, et al (2010) The pneumococcal serine-rich repeat protein is an intra-species bacterial adhesin that promotes bacterial aggregation *in vivo* and in biofilms. PLoS Pathog 6: e1001044. 20714350 10.1371/journal.ppat.1001044 PMC2920850

[8] Holm L, Rosenstrom P (2010) Dali server: conservation mapping in 3D. Nucleic Acids Res 38: W545–549. 20457744 10.1093/nar/gkq366 PMC2896194

[9] Pletnev VZ, Ruzheinikov SN, Tsygannik IN, Mikhailova IY, Duax W, et al (1997) The structure of pea lectin-D-glucopyranose complex at a 1.9 Å resolution. RUSS. J.Bioorganic Chem 23: 469–478.

[10] Rabijns A, Verboven C, Rouge P, Barre A, Van Damme EJ, et al (2001) Structure of a legume lectin from the bark of *Robinia pseudoacacia* and its complex with N-acetylgalactosamine. Proteins 44: 470–478. 11484224 10.1002/prot.1112

[11] Murzin AG, Brenner SE, Hubbard T, Chothia C (1995) SCOP: a structural classification of proteins database for the investigation of sequences and structures. J Mol Biol 247: 536–540. 7723011 10.1006/jmbi.1995.0159

[12] Jin X, Walker MA, Felsovalyi K, Vendome J, Bahna F, et al (2012) Crystal structures of *Drosophila* N-cadherin ectodomain regions reveal a widely used class of Ca^2+^-free interdomain linkers. Proc Natl Acad Sci U S A 109: E127–134. 22171007 10.1073/pnas.1117538108 PMC3271863

[13] Sotomayor M, Weihofen WA, Gaudet R, Corey DP (2012) Structure of a force-conveying cadherin bond essential for inner-ear mechanotransduction. Nature 492: 128–132. 23135401 10.1038/nature11590 PMC3518760

[14] Sharon N, Lis H (1990) Legume lectins—a large family of homologous proteins. FASEB J 4: 3198–3208. 2227211 10.1096/fasebj.4.14.2227211

[15] Loris R, Hamelryck T, Bouckaert J, Wyns L (1998) Legume lectin structure. Biochim Biophys Acta 1383: 9–36. 9546043 10.1016/s0167-4838(97)00182-9

[16] Duverger E, Frison N, Roche AC, Monsigny M (2003) Carbohydrate-lectin interactions assessed by surface plasmon resonance. Biochimie 85: 167–179. 12765786 10.1016/s0300-9084(03)00060-9

[17] MacKenzie DA, Tailford LE, Hemmings AM, Juge N (2009) Crystal structure of a mucus-binding protein repeat reveals an unexpected functional immunoglobulin binding activity. J Biol Chem 284: 32444–32453. 19758995 10.1074/jbc.M109.040907 PMC2781659

[18] Graille M, Stura EA, Housden NG, Beckingham JA, Bottomley SP, et al (2001) Complex between *Peptostreptococcus magnus* protein L and a human antibody reveals structural convergence in the interaction modes of Fab binding proteins. Structure 9: 679–687. 11587642 10.1016/s0969-2126(01)00630-x

[19] Bjorck L (1988) Protein L. A novel bacterial cell wall protein with affinity for Ig L chains. J Immunol 140: 1194–1197. 3125250

[20] Wikstrom M, Drakenberg T, Forsen S, Sjobring U, Bjorck L (1994) Three-dimensional solution structure of an immunoglobulin light chain-binding domain of protein L. Comparison with the IgG-binding domains of protein G. Biochemistry 33: 14011–14017. 10.1021/bi00251a008 7947810

[21] Beckingham JA, Bottomley SP, Hinton R, Sutton BJ, Gore MG (1999) Interactions between a single immunoglobulin-binding domain of protein L from *Peptostreptococcus magnus* and a human *κ* light chain. Biochem J 340 (Pt 1): 193–199. PMC1220237 10229674

[22] Graille M, Harrison S, Crump MP, Findlow SC, Housden NG, et al (2002) Evidence for plasticity and structural mimicry at the immunoglobulin light chain-protein L interface. J Biol Chem 277: 47500–47506. 12221088 10.1074/jbc.M206105200

[23] Berman H, Henrick K, Nakamura H, Markley JL (2007) The worldwide Protein Data Bank (wwPDB): ensuring a single, uniform archive of PDB data. Nucleic Acids Res 35: D301–303. 17142228 10.1093/nar/gkl971 PMC1669775

[24] Hura GL, Menon AL, Hammel M, Rambo RP, Poole FL 2nd, et al (2009) Robust, high-throughput solution structural analyses by small angle X-ray scattering (SAXS). Nat Methods 6: 606–612. 19620974 10.1038/nmeth.1353 PMC3094553

[25] Kukita K, Kawada-Matsuo M, Oho T, Nagatomo M, Oogai Y, et al (2013) *Staphylococcus aureus* SasA is responsible for binding to the salivary agglutinin gp340, derived from human saliva. Infect Immun 81: 1870–1879. 23439307 10.1128/IAI.00011-13 PMC3676022

[26] Holmskov U, Lawson P, Teisner B, Tornoe I, Willis AC, et al (1997) Isolation and characterization of a new member of the scavenger receptor superfamily, glycoprotein-340 (gp-340), as a lung surfactant protein-D binding molecule. J Biol Chem 272: 13743–13749. 9153228 10.1074/jbc.272.21.13743

[27] Srinivas VR, Reddy GB, Ahmad N, Swaminathan CP, Mitra N, et al (2001) Legume lectin family, the ‘natural mutants of the quaternary state’, provide insights into the relationship between protein stability and oligomerization. Biochim Biophys Acta 1527: 102–111. 11479026 10.1016/s0304-4165(01)00153-2

[28] Velloso LM, Svensson K, Schneider G, Pettersson RF, Lindqvist Y (2002) Crystal structure of the carbohydrate recognition domain of p58/ERGIC-53, a protein involved in glycoprotein export from the endoplasmic reticulum. J Biol Chem 277: 15979–15984. 11850423 10.1074/jbc.M112098200

[29] Brasch J, Harrison OJ, Honig B, Shapiro L (2012) Thinking outside the cell: how cadherins drive adhesion. Trends Cell Biol 22: 299–310. 22555008 10.1016/j.tcb.2012.03.004 PMC3385655

[30] Boggon TJ, Murray J, Chappuis-Flament S, Wong E, Gumbiner BM, et al (2002) C-cadherin ectodomain structure and implications for cell adhesion mechanisms. Science 296: 1308–1313. 11964443 10.1126/science.1071559

[31] Krissinel E, Henrick K (2007) Inference of macromolecular assemblies from crystalline state. J Mol Biol 372: 774–797. 17681537 10.1016/j.jmb.2007.05.022

[32] Bensing BA, Lopez JA, Sullam PM (2004) The *Streptococcus gordonii* surface proteins GspB and Hsa mediate binding to sialylated carbohydrate epitopes on the platelet membrane glycoprotein Ibα. Infect Immun 72: 6528–6537. 15501784 10.1128/IAI.72.11.6528-6537.2004 PMC523053

[33] Samen U, Eikmanns BJ, Reinscheid DJ, Borges F (2007) The surface protein Srr-1 of *Streptococcus agalactiae* binds human Keratin 4 and promotes adherence to epithelial HEp-2 Cells. Infect Immun 75: 5405–5414. 17709412 10.1128/IAI.00717-07 PMC2168289

[34] Shivshankar P, Sanchez C, Rose LF, Orihuela CJ (2009) The *Streptococcus pneumoniae* adhesin PsrP binds to Keratin 10 on lung cells. Mol Microbiol 73: 663–679. 19627498 10.1111/j.1365-2958.2009.06796.x PMC2753542

[35] Maurer P, Hohenester E, Engel J (1996) Extracellular calcium-binding proteins. Curr Opin Cell Biol 8: 609–617. 8939653 10.1016/s0955-0674(96)80101-3

[36] Larson MR, Rajashankar KR, Patel MH, Robinette RA, Crowley PJ, et al (2010) Elongated fibrillar structure of a *Streptococcal* adhesin assembled by the high-affinity association of α- and PPII-helices. Proc Natl Acad Sci U S A 107: 5983–5988. 20231452 10.1073/pnas.0912293107 PMC2851892

[37] Gruszka DT, Wojdyla JA, Bingham RJ, Turkenburg JP, Manfield IW, et al (2012) *Staphylococcal* biofilm-forming protein has a contiguous rod-like structure. Proc Natl Acad Sci U S A 109: E1011–E1018. 22493247 10.1073/pnas.1119456109 PMC3340054

[38] Otwinowski Z, Minor W (1997) Processing of X-ray diffraction data collected in oscillation mode. Method Enzymol, Pt A 276: 307–326. 10.1016/S0076-6879(97)76066-X 27754618

[39] Brodersen DE, de La Fortelle E, Vonrhein C, Bricogne G, Nyborg J, et al (2000) Applications of single-wavelength anomalous dispersion at high and atomic resolution. Acta Crystallogr D Biol Crystallogr 56: 431–441. 10739916 10.1107/s0907444900000834

[40] Adams PD, Afonine PV, Bunkoczi G, Chen VB, Davis IW, et al (2010) PHENIX: a comprehensive Python-based system for macromolecular structure solution. Acta Crystallogr D Biol Crystallogr 66: 213–221. 20124702 10.1107/S0907444909052925 PMC2815670

[41] Cowtan K (2006) The Buccaneer software for automated model building. 1. Tracing protein chains. Acta Crystallogr D Biol Crystallogr 62: 1002–1011. 16929101 10.1107/S0907444906022116

[42] Murshudov GN, Vagin AA, Dodson EJ (1997) Refinement of macromolecular structures by the maximum-likelihood method. Acta Crystallogr D Biol Crystallogr 53: 240–255. 15299926 10.1107/S0907444996012255

[43] Emsley P, Cowtan K (2004) Coot: model-building tools for molecular graphics. Acta Crystallogr D Biol Crystallogr 60: 2126–2132. 15572765 10.1107/S0907444904019158

[44] Collaborative Computational Project N (1994) The CCP4 suite: programs for protein crystallography. Acta Crystallogr D Biol Crystallogr 50: 760–763. 15299374 10.1107/S0907444994003112

[45] Hao Q, Gu YX, Zheng CD, Fan HF (2000) OASIS: a computer program for breaking phase ambiguity in one-wavelength anomalous scattering or single isomorphous substitution (replacement) data. J Appl Crystallogr 33: 980–981.

[46] Davis I, Leaver-Fay A, Chen V, Block J, Kapral G, et al (2007) MolProbity: all-atom contacts and structure validation for proteins and nucleic acids. Nucleic Acids Res 35: W375–383. 17452350 10.1093/nar/gkm216 PMC1933162

[47] Laskowski R, Macarthur M, Moss D, Thornton J (1993) Procheck - a Program to Check the Stereochemical Quality of Protein Structures. J Appl Crystallogr 26: 283–291.

[48] Trott O, Olson AJ (2010) AutoDock Vina: improving the speed and accuracy of docking with a new scoring function, efficient optimization, and multithreading. J Comput Chem 31: 455–461. 19499576 10.1002/jcc.21334 PMC3041641

[49] Sanner MF (1999) Python: a programming language for software integration and development. J Mol Graph Model 17: 57–61. 10660911

[50] Valle J, Latasa C, Gil C, Toledo-Arana A, Solano C, et al (2012) Bap, a biofilm matrix protein of *Staphylococcus aureus* prevents cellular internalization through binding to GP96 host receptor. PLoS Pathog 8: e1002843. 22876182 10.1371/journal.ppat.1002843 PMC3410863

[51] Nelson AL, Ries J, Bagnoli F, Dahlberg S, Falker S, et al (2007) RrgA is a pilus-associated adhesin in *Streptococcus pneumoniae* . Mol Microbiol 66: 329–340. 17850254 10.1111/j.1365-2958.2007.05908.x PMC2170534

[52] Jorgensen WL, Chandrasekhar J, Madura JD, Impey RW, Klein ML (1983) Comparison of simple potential functions for simulating liquid water. J Chem Phys 79: 926–935.

[53] Hess B, Kutzner C, van der Spoel D, Lindahl E (2008) GROMACS 4: Algorithms for highly efficient, load-balanced, and scalable molecular simulation. J Chem Theory Comput 4: 435–447. 26620784 10.1021/ct700301q

[54] Duan Y, Wu C, Chowdhury S, Lee MC, Xiong GM, et al (2003) A point-charge force field for molecular mechanics simulations of proteins based on condensed-phase quantum mechanical calculations. J Comput Chem 24: 1999–2012. 14531054 10.1002/jcc.10349

[55] Berendsen HJC, Postma JPM, Vangunsteren WF, Dinola A, Haak JR (1984) Molecular dynamics with coupling to an external bath. J Chem Phys 81: 3684–3690.

[56] Hess B (2008) P-LINCS: A parallel linear constraint solver for molecular simulation. J Chem Theory Comput 4: 116–122. 26619985 10.1021/ct700200b

[57] Essmann U, Perera L, Berkowitz ML, Darden T, Lee H, et al (1995) A smooth particle mesh Ewald method. J Chem Phys 103: 8577–8593.

[58] Humphrey W, Dalke A, Schulten K (1996) VMD: Visual molecular dynamics. J Mol Graph 14: 33–38. 8744570 10.1016/0263-7855(96)00018-5

[59] Konarev PV, Volkov VV, Sokolova AV, Koch MHJ, Svergun DI (2003) PRIMUS: a Windows PC-based system for small-angle scattering data analysis. J Appl Crystallogr 36: 1277–1282.

[60] Svergun DI (1992) Determination of the Regularization Parameter in Indirect-Transform Methods Using Perceptual Criteria. J Appl Crystallogr 25: 495–503.

[61] Svergun DI (1999) Restoring low resolution structure of biological macromolecules from solution scattering using simulated annealing (vol 76, pg 2879, 1999). Biophys J 77: 2896–2896. 10.1016/S0006-3495(99)77443-6 PMC1300260 10354416

[62] Svergun DI, Petoukhov MV, Koch MHJ (2001) Determination of domain structure of proteins from X-ray solution scattering. Biophys J 80: 2946–2953. 11371467 10.1016/S0006-3495(01)76260-1 PMC1301478

