# Supplementary figures and images for: Structural Insights into SraP-Mediated Staphylococcus aureus Adhesion to Host Cells
Source: PLoS Pathog. 2014 Jun 5;10(6):e1004169. doi: 10.1371/journal.ppat.1004169 (PMC4047093; doi:10.1371/journal.ppat.1004169)

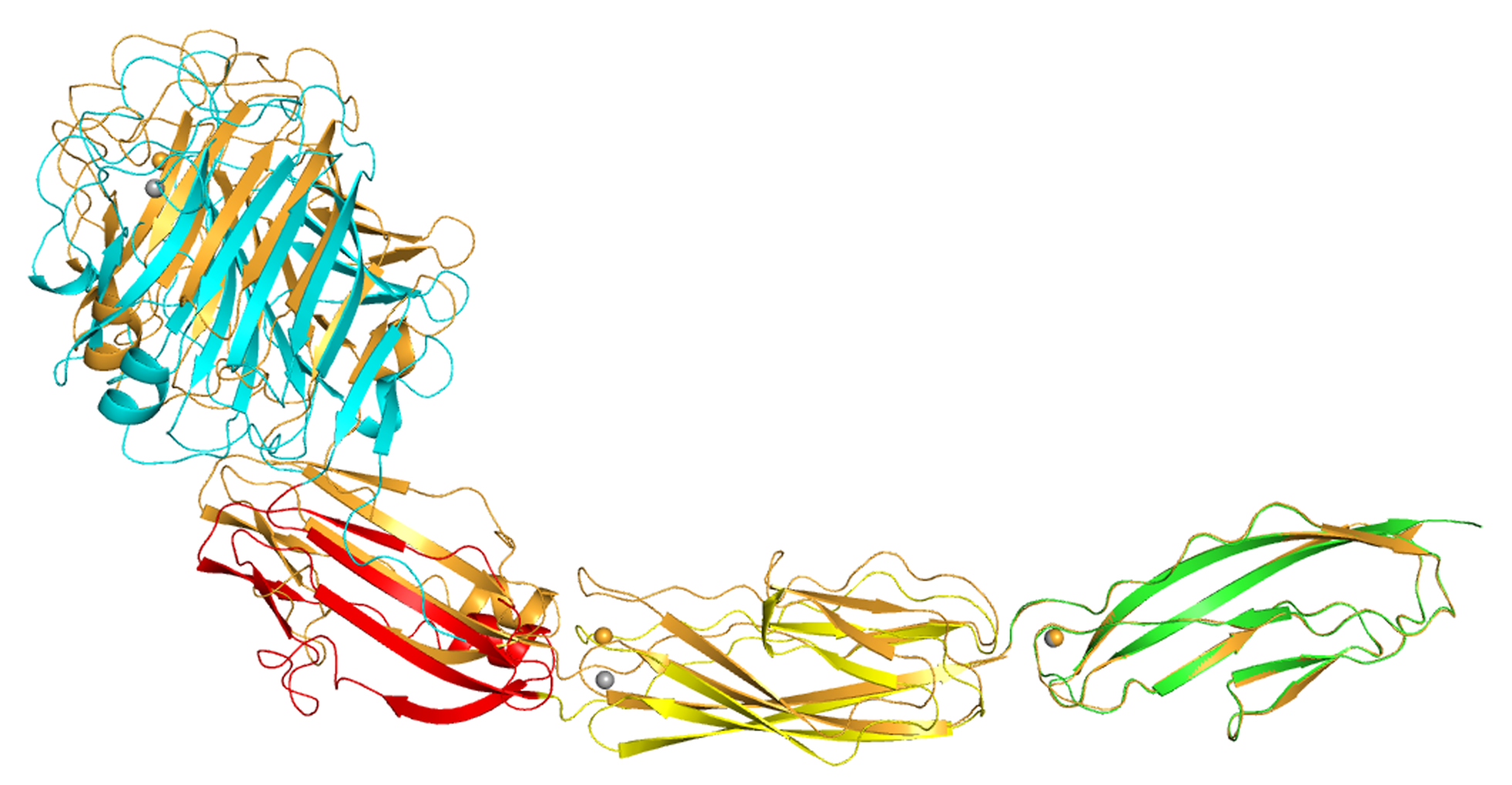

Supplement: Figure S1 — The intermodule twist along the axis of SraPBR. The merged structure of SraPBR (in orange) was generated by sequentially superimposing the same module against each other from the three structures (L-lectin&β-GF, β-GF&CDHL-1 and CDHL-1&2). The L-lectin, β-GF, CDHL-1 and CDHL-2 modules of SraPBR are shown in cyan, red, yellow and green, respectively. The merged SraPBR was superimposed against the crystal structure of SraPBR with the two CDHL-2 modules aligned. (TIF) [file ppat.1004169.s001.tif]

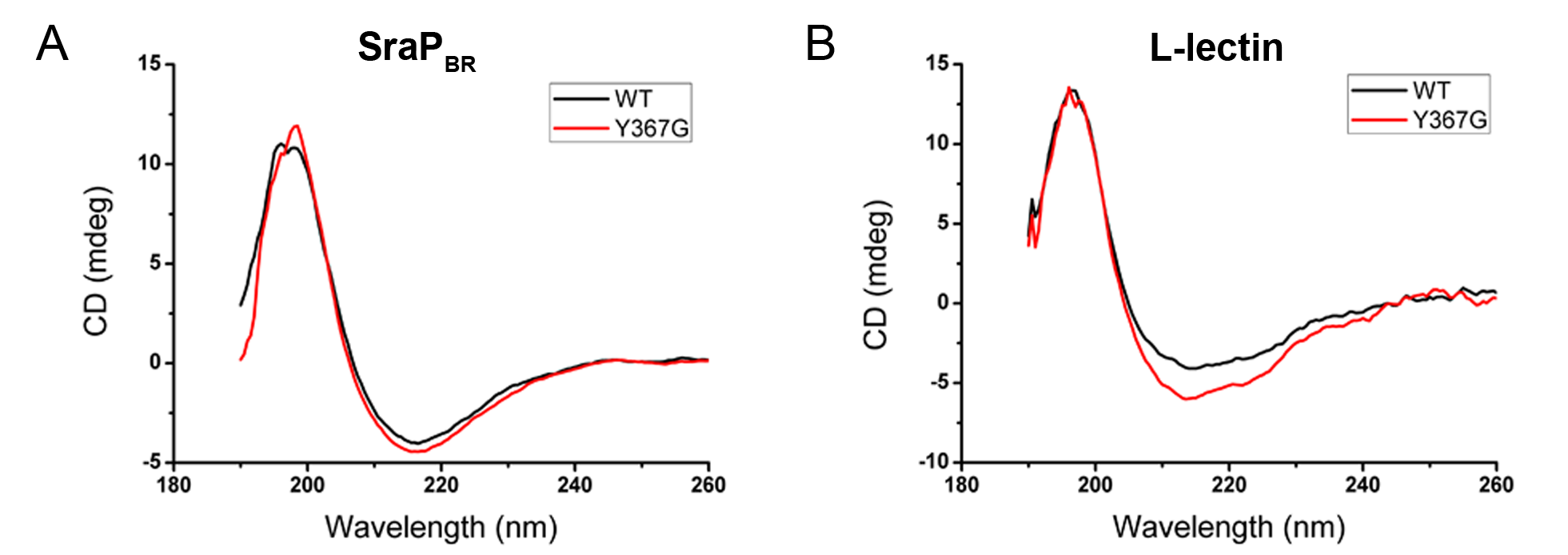

Supplement: Figure S2 — The CD spectra of A) SraPBR and B) the L-lectin module. The results demonstrated that mutation of Y367G did not introduce significant changes to the protein structures. (TIF) [file ppat.1004169.s002.tif]

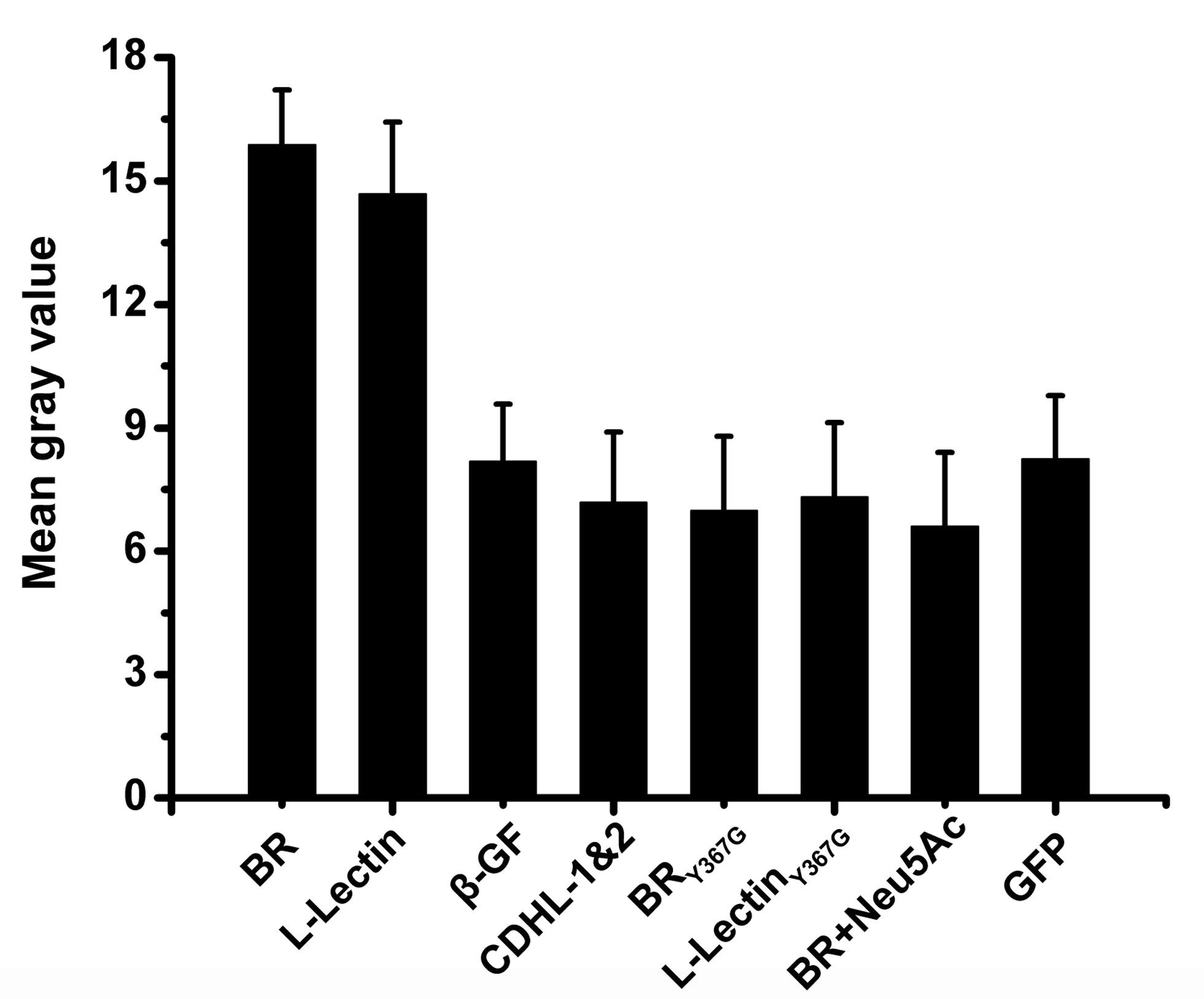

Supplement: Figure S3 — Quantitation of the GFP fluorescent signals of SraPBR and mutants. The fluorescent signals for each protein were quantified by calculating the mean gray values of three representative frames using the ImageJ software (http://imagej.nih.gov/ij/). The average mean gray value and standard error of the mean (SEM) derived from triplicate treatments are indicated as bar graph. (TIF) [file ppat.1004169.s003.tif]

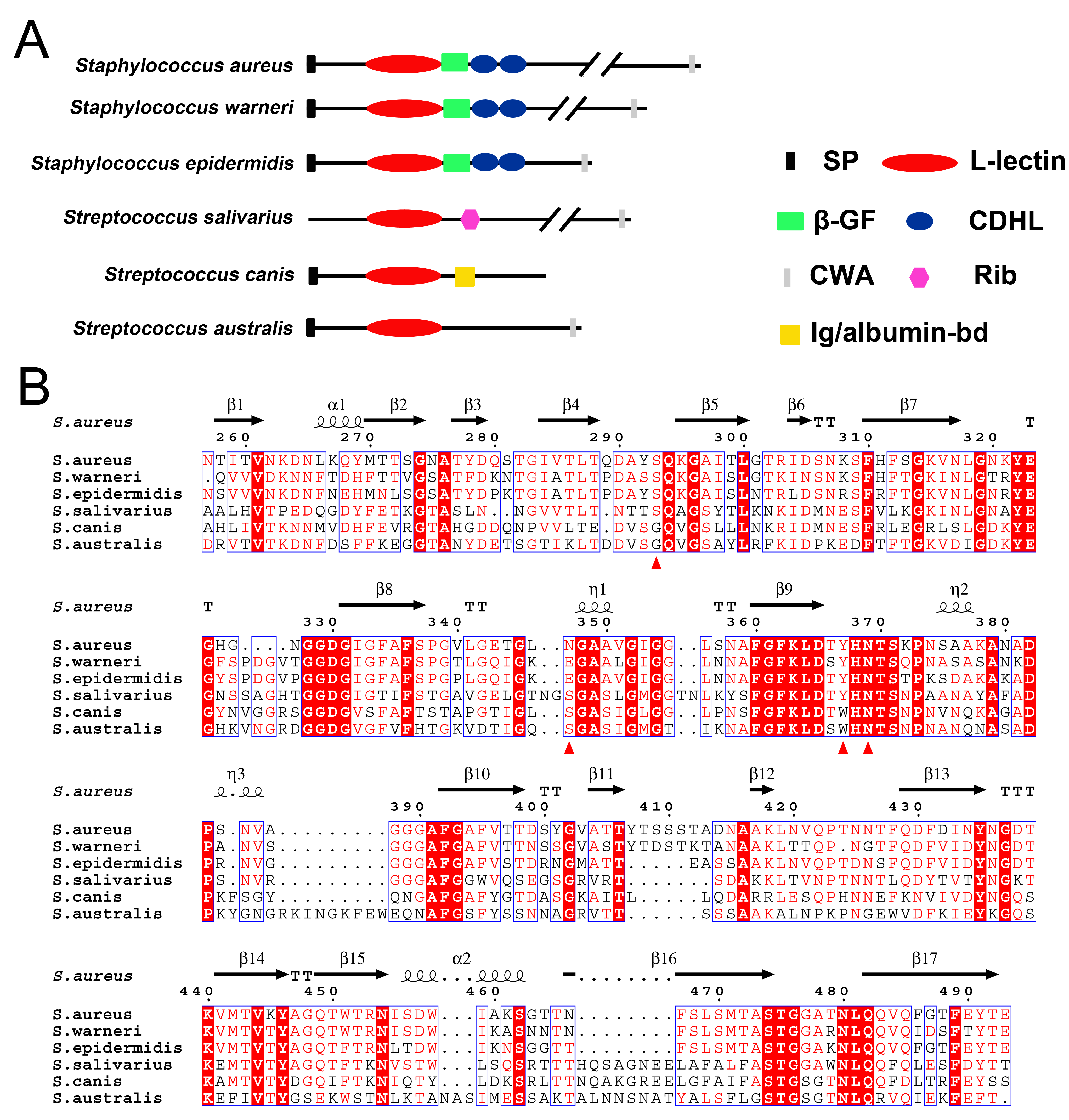

Supplement: Figure S4 — The L-lectin module is conserved in Staphylococci and some species of Streptococci. A) Schematic of Staphylococcal and Streptococcal proteins containing an L-lectin module. The first line represents the SraP protein from S. aureus. Similar modularization is observed in S. epidermidis and S. warneri (2nd and 3rd line). The 4th line represents uncharacterized protein from S. salivarius. The 5th is a serine threonine rich antigen from S. canis, and the last is a putative uncharacterized protein from S. australis. SP: signal peptide, CWA: cell wall anchor motif, Rib: a repeat motif in Rib protein of group B Streptococcus, Ig/albumin-bd: immunoglobulin and albumin-binding domain. B) Multiple-sequence alignment of the L-lectin module and homologs. The Neu5Ac binding residues (red triangle) are conserved. (TIF) [file ppat.1004169.s004.tif]

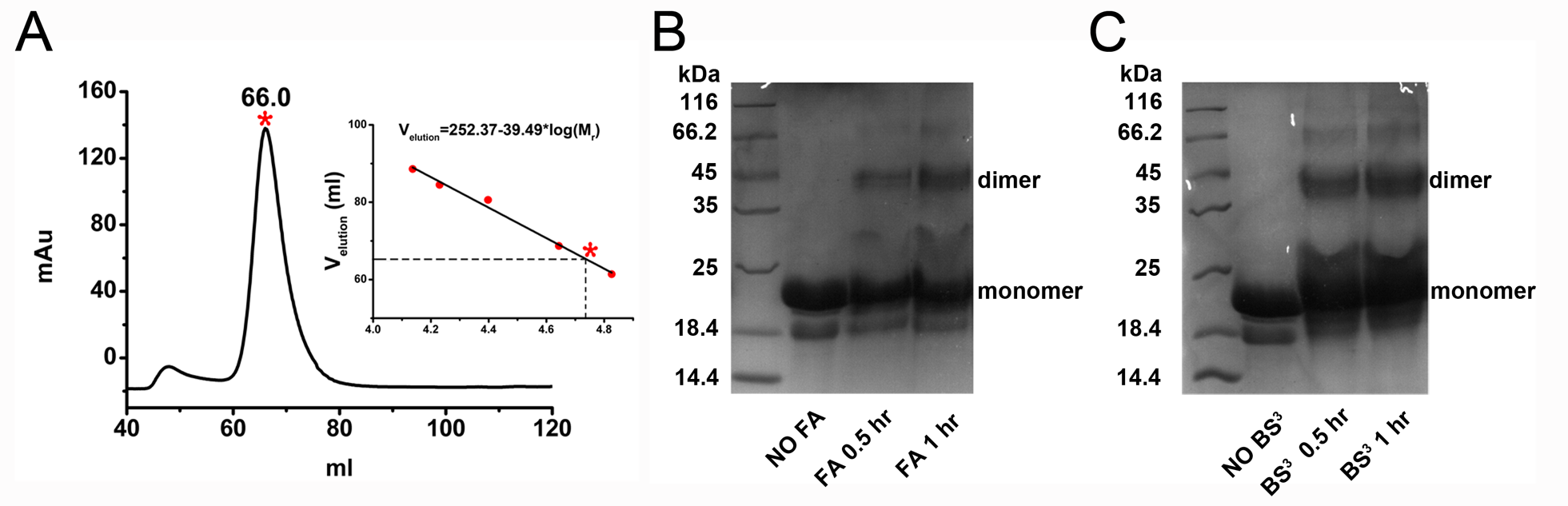

Supplement: Figure S5 — CDHL1&2 exists as a dimer in solution. A) The size-exclusion chromatograph profile of CDHL-1&2. The standard curve was inserted as an inlet. Chemical cross-linking of the purified CDHL-1&2 proteins using B) formaldehyde (FA) and C) bis(sulfosuccinimidyl) suberate (BS3). The protein samples were separated by 10% SDS-PAGE. The bands corresponding to the monomer and the dimer of CDHL1&2 are labeled. (TIF) [file ppat.1004169.s005.tif]
